# Distal Radius Interventions for Fracture Treatment (DRIFT) trial: study protocol for a multicentre randomised clinical trial of completely translated distal radius fractures at paediatric hospitals in North America

**DOI:** 10.1136/bmjopen-2024-088273

**Published:** 2025-10-29

**Authors:** Lauren Balmert Bonner, Joseph Janicki, Andrew Georgiadis, Walter Truong, Dorothy Harris Beauvais, Mohan Belthur, Erika L Daley, Jeanne Franzone, Andrew Howard, Collin May, Frank Rockhold, Jacob Schulz, Mary Bailey, Karen Chiswell, Jesse DeLaRosa, Jaysson T Brooks, Anthony A Cantanzano, Andrea Chan, Alice Chu, Emily R Dodwell, Ron El-Hawary, Henry Ellis, Ryan Fitzgerald, Steven Frick, Theodore J Ganley, Dominic Gargiulo, Luke Gauthier, Corey S Gill, Rachel Goldstein, Matthew F Halsey, Christina Hardesty, Christine Ho, Neil Kaushal, John Todd Lawrence, R Jay Lee, Khristinn K Leitch, Karim Masrouha, Stuart Mitchell, Natasha OMalley, Monica Payares- Lizano, Daniel Perry, Wendy Ramalingam, Jason Rhodes, Julia Sanders, Apurva S Shah, Melinda Sharkey, Mauricio Silva, Selina Silva, Rachael Thompson, John Vorhies, James Gardner Wright, Candace Young, Jamie Burgess, on behalf of IMPACCT Consortium, Pablo G Castaneda

**Affiliations:** 1 Biostatistics Collaboration Center and Northwestern University Data Analysis and Coordinating Center, Northwestern University Feinberg School of Medicine, Chicago, Illinois, USA; 2 Division of Orthopaedics & Sports Medicine, Ann and Robert H Lurie Children’s Hospital of Chicago, Chicago, Illinois, USA; 3 Orthopaedic Surgery, Gillette Children's Specialty Healthcare, Saint Paul, Minnesota, USA; 4 Orthopedics and Sports Medicine, Texas Children’s Hospital, Houston, Texas, USA; 5 Orthopedic Surgery and Sports Medicine, Phoenix Children’s Hospital, Phoenix, Arizona, USA; 6 Pediatric Orthopedic Surgery, Riley Hospital for Children, Indianapolis, Indiana, USA; 7 Department of Orthopedics, Nemours Children's Hopistal, Wilmington, Delaware, USA; 8 Division of Orthopaedic Surgery, Hospital for Sick Children Research Institute, Toronto, Ontario, Canada; 9 Department of Orthopedic Surgery, Boston Children's Hospital, Boston, Massachusetts, USA; 10 Department of Biostatistics and Bioinformatics, Duke University Medical Center and Duke Clinical Research Institute, Durham, North Carolina, USA; 11 Division of Pediatric Orthopedic Surgery, Montefiore- Einstein, Bronx, New York, USA; 12 Duke Clinical Research Institute, Duke University School of Medicine, Durham, North Carolina, USA; 13 Duke Clinical Research Institute, Durham, North Carolina, USA; 14 Department of Orthopaedics, Texas Scottish Rite Hospital for Children, Dallas, Texas, USA; 15 Duke University School of Medicine, Durham, North Carolina, USA; 16 Orthopaedics, The Hospital for Sick Children, Toronto, Ontario, Canada; 17 Pediatric Orthopaedic Surgery, Rutgers Health, Newark, New Jersey, USA; 18 Pediatric Orthopaedics, Hospital for Special Surgery, New York City, New York, USA; 19 Dalhousie University, Halifax, Nova Scotia, Canada; 20 Department of Orthopaedic Surgery, Texas Scottish Rite Hospital for Children, Dallas, Texas, USA; 21 Children's Orthopedic and Scoliosis, Johns Hopkins All Children's Hospital, Tampa, Florida, USA; 22 Stanford Medicine Children’s Health 900 Welch Road, Palo Alto, California, USA; 23 Division of Orthopaedics, Children's Hospital of Philadelphia, Philadelphia, Pennsylvania, USA; 24 Orthopaedic Surgery, Children’s Hospital New Orleans, New Orleans, Louisiana, USA; 25 Orthopaedic Surgery, IWK Health Centre, Halifax, Nova Scotia, Canada; 26 Children's Orthopaedic Center, Children's Hospital Los Angeles, Los Angeles, California, USA; 27 Department of Orthopaedics and Rehabilitation, Oregon Health & Science University, Portland, Oregon, USA; 28 Pediatric Orthopaedic Surgery, University Hospitals Rainbow Babies & Children’s Hospital, Cleveland, Ohio, USA; 29 Orthopaedic Surgery, Scottish Rite for Children, Dallas, Texas, USA; 30 Pediatric Orthopaedics, Johns Hopkins Medicine, Baltimore, Maryland, USA; 31 Orthopaedic Surgery and Rehabilitation, The University of Mississippi Medical Center, Jackson, Mississippi, USA; 32 Orthopedic Surgery, New York University School of Medicine, New York City, New York, USA; 33 Orthopaedics, The University of North Carolina at Chapel Hill Department of Medicine, Chapel Hill, North Carolina, USA; 34 Orthopaedics and Physical Performance, Pediatrics, University of Rochester Medical Center, Rochester, New York, USA; 35 Orthopedic Surgery, Nicklaus Children’s Hospital, Miami, Florida, USA; 36 University of Liverpool, Liverpool, UK; 37 Orthopaedic Surgery, Cincinnati Children’s, Cincinnati, Ohio, USA; 38 Orthopaedic Surgery, Children’s Hospital Colorado, Aurora, Colorado, USA; 39 Orthopaedic Surgery, The Children’s Hospital of Philadelphia, Philadelphia, Pennsylvania, USA; 40 Orthopaedic Surgery, Montefiore Health System, Bronx, New York, USA; 41 Orthopaedic Institute for Children, University of California Los Angeles David Geffen School of Medicine, Los Angeles, California, USA; 42 Orthopaedics and Rehabilitation, University of New Mexico Health Sciences Center, Albuquerque, New Mexico, USA; 43 Pediatric Orthopedic Surgery, Stanford Medicine Children’s Health 900 Welch Road, Palo Alto, California, USA

**Keywords:** ORTHOPAEDIC & TRAUMA SURGERY, Paediatric orthopaedics, Clinical Trial, Randomized Controlled Trial

## Abstract

**Introduction:**

Distal radius fractures are the most common fractures seen in the emergency department in children in the USA. However, no established or standardised guidelines exist for the optimal management of completely displaced fractures in younger children. The proposed multicentre randomised trial will compare functional outcomes between children treated with fracture reduction under sedation versus children treated with simple immobilisation.

**Methods and analysis:**

Participants aged 4–10 years presenting to the emergency department with 100% dorsally translated metaphyseal fractures of the radius less than 5 cm from the distal radial physis will be recruited for the study. Those patients with open fractures, other ipsilateral arm fractures (excluding ulna), pathologic fractures, bone diseases, or neuromuscular or metabolic conditions will be excluded. Participants who agree to enrol in the trial will be randomly assigned via a minimal sufficient balance algorithm to either sedated reduction or in situ immobilisation. A sample size of 167 participants per arm will provide at least 90% power to detect a difference in the primary outcome of Patient-Reported Outcomes Measurement Information System Upper Extremity computer adaptive test scores of 4 points at 1 year from treatment. Primary analyses will employ a linear mixed model to estimate the treatment effect at 1 year. Secondary outcomes include additional measures of perceived pain, complications, radiographic angulation, satisfaction and additional procedures (revisions, refractures, reductions and reoperations).

**Ethics and dissemination:**

Ethical approval was obtained from the following local Institutional Review Boards: Advarra, serving as the single Institutional Review Board, approved the study (Pro00062090) in April 2022. The Hospital for Sick Children (Toronto, ON, Canada) did not rely on Advarra and received separate approval from their local Research Ethics Board (REB; REB number: 1000079992) on 19 July 2023. Results will be disseminated through publication in peer-reviewed journals and presentations at international conference meetings.

**Trial registration number:**

NCT05131685.

**STRENGTHS AND LIMITATIONS OF THIS STUDY**

- This trial will leverage the Infrastructure for Musculoskeletal Pediatric Acute Care Clinical Trials consortium, recruiting participants from over 34 clinical sites across the USA and Canada.
- The randomised pragmatic study design will mirror typical clinical visits and leverage electronic consent and data collection, minimising the burden on surgeons and participants.
- Centralisation of study activities through clinical and data coordinating centres will increase efficiency in identifying, consenting and following participants.
- Participants will be randomised centrally by the Data Coordinating Center via a minimal sufficient balance algorithm to prevent serious imbalances between treatment arms on centre, age and sex.
- This is an open unmasked trial as participants and surgeons are not blinded to individual interventions at their sites.

## Introduction

 Distal radius fractures (DRFs) make up 20–25% of all paediatric fractures^1 2^ and are the most common fractures seen in the emergency department in children in the USA.^3^ The available evidence on DRF reduction/non-reduction is based on case series, observational comparisons and expert opinions. Displaced, bayonetted metaphyseal DRFs have historically been treated with attempts at closed reduction (under conscious sedation or anaesthesia). This approach was supported by retrospective studies and consensus opinion that anatomical alignment was necessary for functional range of motion.^4 5^ Furthermore, it is unsettling for physicians and families to see bones overlapped on a radiograph when a reduction procedure can be completed in a straightforward fashion. However, simple immobilisation without attempted reduction has been reported for management of DRFs in children under age 10.^6^ This approach is conceptually supported by the fracture’s proximity to the distal radial physis and the remaining growth of the child, which provides significant remodelling potential and can allow for improvement of malalignment as the child grows.^6 7^ There is a paucity of literature comparing reduced to non-reduced fractures to guide management. No established or standardised guidelines exist for these types of fractures.

Surveys have identified widely discrepant recommendations and high practice variation for treatments for overlapped DRF patterns.^8^ In the UK, surgical pin fixation is used routinely for fears of reisplacement.^9 10^ In the USA, identical injuries would undergo different treatment depending on the surgeon’s background (hand surgeon, paediatric orthopaedist, general orthopaedist or orthopaedic traumatologist), and depending on the practice model (public vs private).^11^

Although existing studies provide preliminary data to support clinical management, the studies lack a control population for comparison, are retrospective, lack randomisation, have variable follow-up times and/or have no standard definitions of outcomes. In addition, the studies used radiographic or non-validated outcome measures to make conclusions, limiting their utility in identifying optimal management. Previous trials of distal radius fractures in children often involved surgical fixation, a focus on long arm versus short arm casts, and none had patient-reported outcomes.^1012^,^15^

Thus, the proposed trial will compare the effectiveness of alignment under sedation/anaesthesia versus in situ immobilisation for management of overriding DRFs in children, providing critical data regarding optimal management of this common fracture. Therefore, this study’s primary question is the following: does anatomic reduction under sedation/anaesthesia of DRF result in different patient outcomes at 1 year compared with immobilisation without attempted reduction? Secondary objectives include (1) quantifying differences in perceived pain, (2) evaluating cosmetic differences, (3) quantifying differences in complication rates, (4) estimating cost differences, (5) determining patient and parent satisfaction and return to activities and (6) evaluating long-term functional differences.

## Method and analysis

### Study design

**Figure 1:**
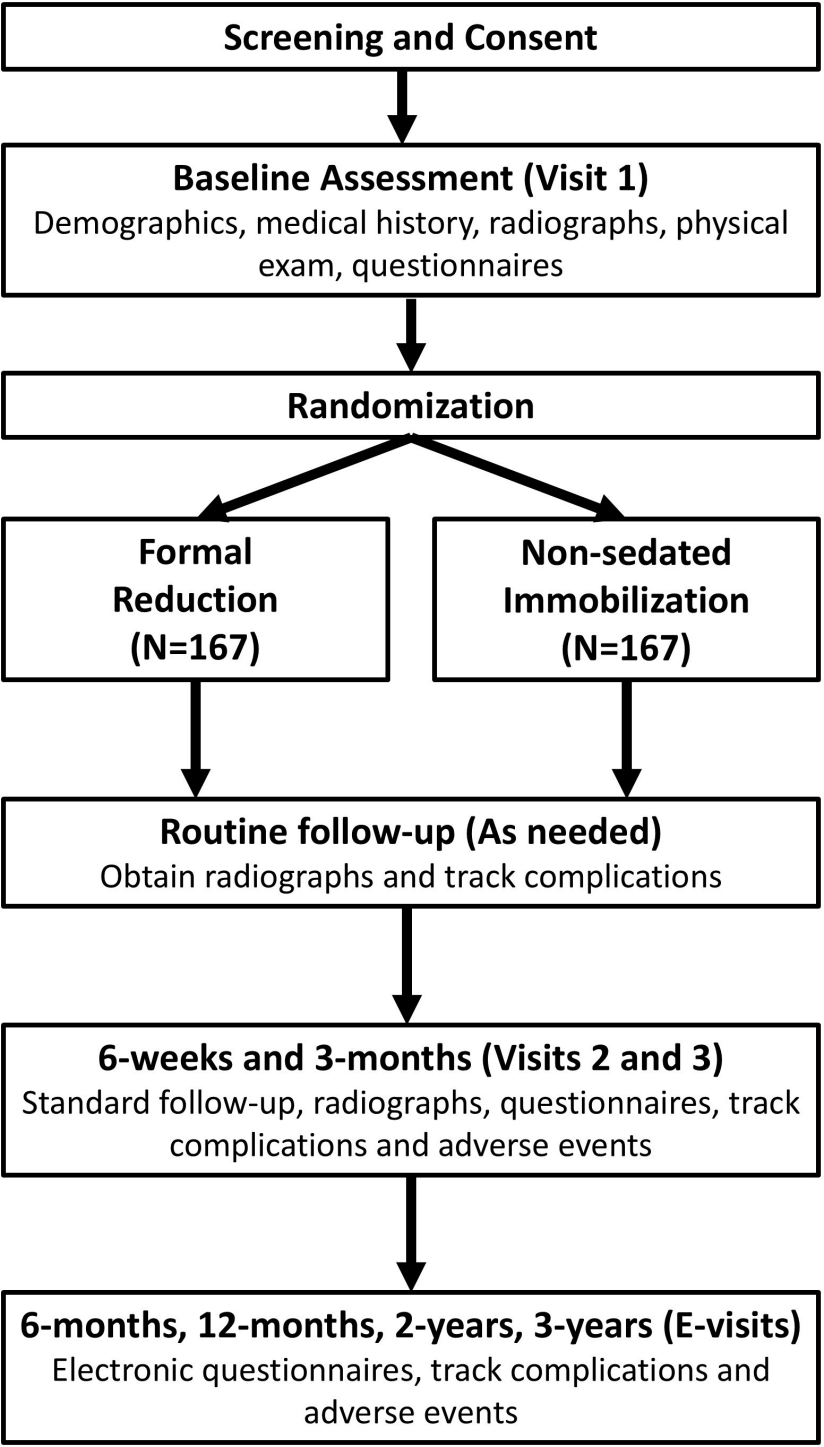
Study schema.

This study is a multicentre, parallel randomised (1:1) controlled trial evaluating two common treatments for completely displaced, overriding metaphyseal DRFs. Designed to avoid large interruptions in busy orthopaedic practices or emergency departments, the practical design will mirror clinical care. In-person assessments will be conducted at baseline, 6 weeks and 3 months to include physical exams, questionnaires and radiographic or other imaging assessments. Anterior-posterior and lateral radiographs of the wrist and forearm will be obtained, and complications will be tracked. Subsequent collection of questionnaires at 6 months, 1 year, 2 years and 3 years will leverage electronic surveys to reduce participant and clinical staff burden (figure 1). Additional radiographs will be collected for any participant returning to the clinic during the follow-up period. Participants will complete all study activities 3 years after fracture.

Given the nature of the intervention, it is not practical for patients or treating surgeons to be blinded to the randomised treatment group. In order to reduce bias from the lack of blinding to treatment, the treating surgeon will not provide primary outcome follow-up data on the patient nor participate in the final analysis. Patient-reported outcome surveys will be administered by separate personnel from the care team, and follow-up data will be collected directly from the patient and family.

The Distal Radius Interventions for Fracture Treatment trial is governed by executive and steering committees. The executive committee is responsible for overall study leadership and oversight (principal investigator: Dr. Janicki). The steering committee includes all members of the executive committee and additional physician-representatives from participating clinical centres and the Data Coordinating Center (DCC). The steering committee is responsible for the conduct of the trial and provided scientific input on study design and implementation. The DCC, led by the Duke Clinical Research Institute, will coordinate data management, data monitoring and statistical analyses. The Clinical Coordinating Center (CCC), located at Lurie Children’s Hospital, will provide overall project management, regulatory, training and monitoring support. Centralised recruiting and dedicated follow-up by research personnel at the CCC, along with remuneration for visit completion, will facilitate enrolment and minimise attrition.

### Participants

Patients will be recruited from the emergency departments or the orthopaedic clinics in the Infrastructure for Musculoskeletal Pediatric Acute Care Clinical Trials (Distal Radius Interventions for Fracture Treatment (DRIFT) trial: study protocol for a multicentre randomised clinical trial in children) consortium. The Infrastructure for Musculoskeletal Pediatric Acute Care Clinical Trials consortium includes 34 hospitals across the USA and Canada, recruited from members of the Pediatric Orthopaedic Society of North America. Information on participating sites can be found on the ClinicalTrials.gov record (online supplemental file 1). Patients aged 4–10 inclusive who present to the emergency department or the orthopaedic clinics with 100% dorsally translated metaphyseal fractures of the radius will be assessed for inclusion by site staff trained in the protocol (AO PCCF classification 23-M/3.1 and 23r-M/3.1 are included).^16^ If the family is interested in learning more about the study, orthopaedic or emergency department representatives will provide the family with an iPad so they can view a short informational video (https://drift-study.digitrial.com) and read more about the study. If the family is interested, they can discuss the study with the CCC via videoconference. The CCC is staffed by research personnel and will be available 24 hours/day to facilitate enrolment, discuss study information and consent process (see online supplemental files 2; 3), and answer any questions with patient/family in real time. If the family is interested in enrolling, either the orthopaedic attending/representative or the CCC will instruct them on how to begin the e-consent process. The family will then consent via the clinic iPad or personal device and only one parent/guardian is required for consent. If the child is between the ages of 7 and 10 years old, assent is obtained. Once the e-consent form is completed, and the assent form if applicable, the family will be provided a copy of the form. If the patient and family decline study participation, the patient will be treated per the treating physician’s regular care recommendation. If consent cannot be obtained from a parent or legal representative, then the patient is not approached for participation. Children and families who are approached for enrolment and decline will be added into a screening log. The screening log will include demographic information including age, sex, race, ethnicity, who approached the patient, and, if they decline participation, reason why they declined and their ultimate treatment.

Inclusion and exclusion criteria are detailed in box 1.

Box 1 **DRIFT inclusion and exclusion criteria**

**Inclusion criteria**

- Provision of signed and dated informed consent form by parent or legal guardian.
- Stated willingness to comply with all study procedures and availability for the duration of the study.
- Male or female, aged 4–10 years inclusive.
- Diagnosis of 100% dorsally translated metaphyseal fractures of the radius with any or no distal ulna involvement near the same level in the ipsilateral arm (AO Classification of Long Bone Fractures 23-M/3.1, 23-M/3.2, 23rM/3.1, 23rM/3.2).
- Fracture is less than 5 cm from the distal radial growth plate.
- Fracture is acute (occurred less than 10 days prior to consent and assignment of treatment arm AND with ability to be taken to operating room (OR) or reduced in the emergency department (ED)).
- Willing to adhere to the immobilisation regimen.

**Exclusion criteria**

- Physeal involvement of fracture.
- Presence of fractures other than the ulna near the same level in the ipsilateral arm.
- Presence of pathologic fracture or open fracture.
- Metabolic or neuromuscular diagnosis, or bone disease.
- Patient and parent or legal guardian are unable to adhere to procedures or complete follow-up due to insufficient comprehension of consent form or surveys or developmental delay.
- Patient and parent or legal guardian not fluent in English or Spanish.
- Patient is a ward of the state.

### Interventions

The study interventions investigated herein are a direct comparison of reduction under sedation of paediatric distal radius fractures versus simple immobilisation. Based on a recent survey study, reduction under sedation is the most commonly employed and recommended treatment pathway, likely due to concerns for long-term functional consequences due to fixed deformity.^8^ Simple immobilisation without attempted reduction is often employed by a significant portion of surgeons surveyed based on previous studies showing significant remodelling potential of these overriding fractures and no difference in functional outcomes at 1 year after injury. Simple immobilisation avoids the need for sedation and avoids the risk of re-displacement, which may occur after reduction. Comparison of the two treatments does not involve investigational drugs, biologic pharmaceuticals or devices. A reduction procedure is accompanied by various levels of deep sedation in the emergency department or operating room setting. For immobilisation without reduction, surgeons may provide pain medication to facilitate the casting procedure. Analgesia should seek to enhance the comfort of the child, though should not intentionally alter their conscious level/responsiveness using the American Association of Anesthesiologists Definition of General Anesthesia and Levels of Sedation/Analgesia.

Both treatment arms involve one-time administration of a fracture treatment by a physician or physician extender. Adherence to the intervention will be defined as receiving only the treatment procedure to which the participant was originally randomised. Non-adherence or cross-over for this study is defined as when a patient is randomised to one treatment arm but receives the procedure for the other. Concomitant medications, supplements, complementary or alternative therapies, treatments or procedures will be allowed after participant randomisation to one of the two treatment arms with the approval of the treating surgeon. This means that later surgery, subsequent manipulation or any other care decision is completely unconstrained by the trial and will be recorded. Medications or therapies that a patient is undergoing prior to the injury are allowed to continue. The type of immobilisation is per surgeon discretion and will be documented. Immobilisation can include long arm cast, short arm cast and splints per the surgeons’ discretion. Any changes in immobilisation or additional procedures will be documented and analysed in the post hoc analysis.

### Randomisation

In an attempt to reduce serious imbalance in baseline covariates believed to be prognostic of the primary outcome and preserve treatment allocation randomness, patients will be randomised via a minimal sufficient balance (MSB) algorithm.^17^ Briefly, when the next participant is to be randomised, distributions of baseline covariates from previously enrolled participants will be compared between treatment arms. The three variables included in the MSB algorithm will be hospital site, sex and age (continuous). A voting scheme, incorporating measures of imbalance for all covariates of interest, will determine whether the next participant is randomised with simple randomisation or with a biased coin assignment. If any covariate crosses the threshold for imbalance and randomising the participant to a particular arm will reduce imbalance, the participant will be randomised with a biased coin probability to that arm. As participants need to be randomised in real time, randomisation will occur through the Research Electronic Data Capture (REDCap) platform managed by the DCC. Data entry triggers will be used to execute an external SAS program to pull data from the REDCap project, run the MSB algorithm and assign the participant to an arm. The SAS program will then push the assignment back into REDCap.

### Sample size considerations

Sample size calculations were based on detecting a clinically meaningful difference in the Patient-Reported Outcomes Measurement Information System (PROMIS) Pediatric Computer Adaptive Test (CAT) V.2.0 - Physical Function - Upper Extremity (UE) of 4 points. PROMIS measures use a T-score metric with a mean of 50 and SD of 10 in a reference population.^18^ A sample size of 133 per arm, assuming a two-sided type I error rate of 0.05, will provide 90% power to detect a difference between arms of 4 points. To conservatively account for 20% lost to follow-up or missing data on the primary outcome at 12 months, we have inflated our sample size to 167 per arm, for a total target enrolment of 334.

### Interim analysis

No interim analyses are planned for efficacy or futility. An interim analysis for sample size re-estimation is planned to allow for modifications to the planned sample size, accounting for uncertainty in the initial design assumptions. Specifically, a blinded sample size re-estimation based on the SD of the primary outcome will be performed after the first 100 participants have completed the 6-month follow-up. The DCC unblinded statistical team will obtain an estimate of the within-group SD using the 6-month assessment of the primary outcome. The decision to increase the sample size, if the observed SD is larger than the SD assumed during trial planning, will be based only on the preliminary estimate of the SD and not on preliminary estimates of treatment effect. This approach to sample size re-estimation allows for sample size adjustment based on the observed interim variance compared with the pretrial variance, without affecting type I error. The target enrolment may be increased accordingly, after review by the steering committee. No formal interim analyses for efficacy or futility will be planned. The Data and Safety Monitoring Board (DSMB) will review data summarisations on adverse events and primary response variables both overall and by study arm. The DSMB may request additional formal interim safety summaries for harm to help inform decisions and recommendations. There will be no formal stopping guidelines and decisions to suspend or discontinue the trial will be based on DSMB recommendations.

### Outcomes

The primary outcome of interest is the PROMIS Pediatric CAT – Upper Extremity measured at 1 year (table 1). The measure is a validated patient-reported outcome for function that is correlated with physiological tests of function.^18^ Secondary outcomes of interest include additional measures of perceived pain captured by the Disabilities of the Arm, Shoulder and Hand – Sports / Performing Arts Module (DASH S/PA), PROMIS Pediatric V.2.0 - Pain Interference, Wong-Baker FACES pain score, and PROMIS Pediatric V.1.0 Global Health (7+2). Cosmesis (assessed by parent rating of appearance), complications, missed work (parents) or school, parent-reported satisfaction, radiographic angulation, and occurrence/number of revisions, refractures, re-reductions and reoperations will also be assessed.

**Table 1:** Outcome measures and time points of analysis

	Measure	Time-point	
Primary	PROMIS Pediatric CAT V.2.0 – Physical Function – Upper Extremity^19^	1 year	
Secondary	PROMIS Pediatric CAT V.2.0 – Physical Function – Upper Extremity	6 weeks, 3 months, 6 months	
	DASH – S/PA^20^	6 weeks, 3 months, 6 months, 1 year*	
	PROMIS Pediatric V.2.0 - Pain Interference^19^	6 weeks, 3 months*, 1 year	
	Wong-Baker FACES Pain score^21^	6 weeks, 3 months*	
	PROMIS Pediatric V.1.0 - Global Health (7+2)^22 23^ – Global Score	6 weeks, 3 months, 6 months, 1 year*	
	PROMIS Pediatric V.1.0 - Global Health (7+2)^22 23^ – Fatigue interference	6 weeks, 3 months, 6 months, 1 year	
	PROMIS Pediatric V.1.0 - Global Health (7+2)^22 23^ – Pain interference	6 weeks, 3 months, 6 months, 1 year	
	Number of revisions, refractures, reductions and reoperations	1 year*	
	Other complications	1 year	
	Missed work or school	3 months*	
	Satisfaction questionnaire^6^	3 months, 6 months*, 1 year	
	Cosmesis	6 weeks, 3 months, 6 months, 1 year*	
	Radiographic alignment	6 weeks†	

* Indicates time point of clinical interest incorporated in family of hypothesis tests with adjustment for multiplicity.

† Radiographic is only collected as part of standard of care and is only mandated at the 6-week visit.

CAT, Computer Adaptive Test; DASH – S/PA, Disabilities of the Arm, Shoulder and Hand – Sports/Performing Arts Module; PROMIS, Patient-Reported Outcomes Measurement Information System.

The statistical methods detailed here focus on analyses incorporating outcomes measured through 1 year. Exploratory objectives beyond 1 year will be detailed elsewhere.

### Analysis populations and estimands

Primary analyses will be based on an intention-to-treat (ITT) principle (treatment policy estimand) and will include all participants randomised. For the treatment policy estimand, participants will be analysed according to their randomised treatment assignment. Anticipated intercurrent events include treatment cross-over, withdrawal from study or lost to follow-up. Outcomes collected after treatment cross-over will be included in the analysis regardless of this intercurrent event, to align with the ITT analysis. Outcomes that are missing due to withdrawal, lost to follow-up or other reasons will be handled as described below. A sensitivity analysis will be based on a per-protocol population, to assess the robustness of the ITT analysis. The per-protocol (adherence estimand) population will be defined as all randomised participants who receive their intended intervention. If a participant crosses over to the alternative treatment during the follow-up period, this participant will be included in their allocated arm, but follow-up will be censored at the time of treatment cross-over. Thus, only outcomes collected prior to treatment cross-over will be included.

### Statistical analyses

Descriptive statistics will summarise demographics, medical history, baseline outcome measures and other clinical characteristics by arm. The participant disposition will be summarised using frequencies and proportions for all ITT participants. The study will follow Consolidated Standards of Reporting Trials guidelines for reporting results.

Analysis for the primary aim will use a mixed-effect model for repeated measures of the primary outcome, PROMIS Pediatric CAT – Upper Extremity, measured at baseline (reflecting preinjury status), 3 months, 6 months and 1 year. The model will include fixed effects for treatment arm, time as a categorical variable, and the treatment arm by time interaction term. Sex and age (continuous) will be included as covariates. Incorporation of a random centre effect will allow for separation of between-site and within-site variance components. For the repeated measures within a participant, we will assume an unstructured covariance matrix. If the model does not converge, then a simpler covariance structure (compound symmetry) will be used. We will report estimates for the mean and SD at 1 year for each arm. The between-arm difference in model-estimated 1 year means will be reported with corresponding 95% CI and p value. The primary analysis will be assessed using a two-sided type I error rate of 0.05, with no adjustment for testing of secondary or exploratory outcomes. Two sensitivity analyses will be conducted including a per-protocol analysis and analyses using multiple imputation for missing data, as described below. Model validity will be assessed via residual diagnostics. We will graphically assess the distribution of residuals to determine if there is gross departure from normality, and if so, transformations may be considered. The unstructured covariance matrix allows for differential variance across time points. If there is evidence of a differential variance between treatment arms, then the covariance matrix may be estimated separately for each arm.

Secondary analyses will incorporate a similar modelling strategy for all continuous repeated measures outcomes. A generalised linear mixed-effect (GLMM) model will be fit for binary and count outcomes with appropriate distributional assumptions and link functions. Specifically, Poisson loglinear GLMM models will be fit for the number of missed school days and number of missed work days of the parent(s). Logistic GLMM models will be fit for parent satisfaction, defined as parents who reported being at least somewhat satisfied, and for occurrence of revisions, refractures, re-reductions and/or reoperations. A false discovery rate correction will be applied to account for multiplicity within the prespecified family of hypothesis tests at key clinical time points for each secondary outcome (table 1). P values for additional time points and all additional exploratory analyses will not be adjusted for multiplicity.

Planned subgroup analyses of the primary outcome may include assessment of treatment effects by age group and sex. Specifically, models will be fit with interactions between the treatment arm and each of these variables. Although we anticipate being underpowered for these specific interactions, these models will be used to assess whether there is evidence that the effect of the intervention is different depending on these prespecified subgroups.

### Missing data

Every effort will be made to minimise missing data for assessment in participants who have not withdrawn from the study. For the main analysis, we will use a mixed model for repeated measures analysis, including all available measurements of the response variable collected at 3 months, 6 months and 1 year as well as the fixed and random effects specified above. This model assumes that data are missing at random, conditional on the measurements included in the model.

Mechanisms of missing data will be examined, and if appropriate, sensitivity analyses will be performed using multiple imputation methods. Specifically, we will use a fully conditional specification for linear mixed models method to impute incomplete longitudinal outcomes. This approach assumes that missingness is at random, conditional on the variables included in the imputation model. Should there be reason to believe the data are not missing at random, sensitivity analyses will be considered with multiple imputations using the delta-adjustment approach.

### Data management

Clinical data and patient-reported outcomes will be entered into REDCap, a 21 CFR Part 11-compliant data capture system. The data system includes password protection and internal quality checks, such as automatic range checks, to identify data that appear inconsistent, incomplete or inaccurate. Clinical data will be entered directly from the source documents.

### Patient and public involvement

As part of a National Institutes of Health (NIH) planning grant (R34), patients and parents responded to questionnaires in a simulated recruitment setting. Input from these patients and parents was used to refine recruitment methods and estimate recruitment rates.

## Ethics and dissemination

Safety oversight will be under the direction of a DSMB composed of individuals with the appropriate expertise and knowledge of paediatric orthopaedic surgery or of clinical trial design and analysis. Members of the DSMB are independent from the study conduct and free of conflicts of interest. The DSMB will meet at least semiannually to assess safety data on each arm of the study. As this is a minimal-risk study in healthy children, there is no study drug or device, and all treatments are considered standard of care, adverse events (AEs) or serious AEs are not expected. However, there is potential for a participant to experience negative outcomes relating to fracture healing. Such negative outcomes included, but are not limited to, malunion or non-union of the fracture, refracture, infection or incomplete healing. Such negative outcomes will be captured in the database. Incidence reports of these outcomes will be provided to the DSMB through their regular reporting schedule as outlined in the Data Safety Monitoring Plan. The DSMB will also monitor overall trial conduct including enrolment, retention and site performance.

Participant confidentiality and privacy are strictly held in trust by the participating investigators, their staff and the sponsor. The study participant’s contact information will be securely stored at each clinical site for internal use during the study. Study participant research data, which is for purposes of statistical analysis and scientific reporting, will be transmitted to and stored at the DCC. This will not include the participant’s contact or identifying information. Rather, individual participants and their research data will be identified by a unique study identification number. The study data entry and study management systems used by clinical sites and by DCC research staff will be secured and password protected.

The trial will comply with the NIH Data Sharing Policy and Policy on the Dissemination of NIH-Funded Clinical trials. Results of the study will be disseminated in peer-reviewed manuscripts, professional conference presentations and reported on ClinicalTrials.gov. The trial was registered on ClinicalTrials.gov in November 2021. Enrolment began in April 2023 and is expected to be completed by April 2026. At the time of publication, the trial is using protocol V.6.0; 15 June 2022. Ethical approval for this study was provided by Advarra Institutional Review Board (Pro00062090), serving as the single IRB of record. The following local Institutional Review Boards participated via reliance agreements with Advarra:

- Lurie Children’s Hospital, Chicago, IL. 9 May 2022.
- Gillette Children’s Hospital, St Paul, MN. 11 October 2022.
- Phoenix Children’s Hospital, Phoenix, AZ. 10 August 2022.
- Cincinnati’s Children’s Hospital, Cincinnati, OH. 5 October 2022.
- Johns Hopkins Children’s Hospital, Baltimore, MD. 9 August 2022.
- Children’s Hospital of Los Angeles, Los Angeles, CA. 4 October 2023.
- Texas Scottish Rite, Dallas, TX. 11 July 2023.
- Children’s Hospital Boston, Boston, MA. 14 October 2022.
- University of Rochester Medical Centre, Rochester, NY. 3 June 2024.
- Niklaus Childrens Hospital, Miami, FL. 26 April 2024.
- UNM Carrie Tingley Hospital, Albuquerque, NM. 24 July 2024.
- Texas Children’s Hospital, Houston, TX. 15 September 2022.
- Case Western Reserve University, Cleveland, OH. 3 March 2023.
- University of Mississippi Medical Centre, Jackson, MS. 21 July 2022.
- Hospital for Special Surgery, New York, NY. 9 August 2023.
- Montefiore Medical Centre, Bronx, NY. 2 March 2023.
- Riley Hospital for Children, Indianapolis, IN. 14 July 2022.
- Oregon Health & Science University, Portland, OR. 28 September 2022.
- University of California at Los Angeles, Los Angeles, CA. 18 August 2023.
- New York University – Langone Health, New York, NY. 22 September 2023.
- University of North Carolina, Chapel Hill, NC. 9 November 2022.
- Rutgers University, New Brunswick, NJ. 2 October 2023.
- Children’s Hospital of Philadelphia, Philadelphia, PA. 10 October 2022.
- Children’s Hospital New Orleans, New Orleans, LA. 20 September 2023.
- Seattle Children’s Hospital, Seattle, Washington. 12 September 2024.

The Hospital for Sick Children (Toronto, ON, Canada) did not rely on Advarra and received separate approval from their local Research Ethics Board (REB; REB number: 1000079992) on 19 July 2023.

## Discussion

Large gaps remain in understanding some of the most common musculoskeletal injuries in children. We expect that the results of this study will clarify the functional and patient-reported outcomes of completely displaced distal radius fractures in a way that has not been hitherto achieved.

Our multicentre trial includes many improvements over previously published, smaller investigations. The trial will include multiple centres and therefore include many populations and different treatment paradigms. Therefore, we anticipate the results to be widely generalisable. The focus on patient-reported outcomes as primary aims in the study will be in distinction to the existing literature, which has historically focused on surrogate-reported or surgeon-reported outcomes, or non-systematic satisfaction of families which may be susceptible to recall bias or social desirability bias (a desire to align responses with what a patient perceives a caregiver wants to hear).

Although the methods have been carefully designed, there are limitations to the study design. Recruitment has been simulated with an antecedent grant and study and has been deemed to be feasible, but real-world enrolment and willingness to randomise may differ from simulated recruitment. There are practical realities to orthopaedic referral that include counselling by outside medical providers and might increase the likelihood of a family having the predisposition to one treatment arm or another. Enrolment data will be tracked and published for transparency. Some patients may not have in-person follow-up after the initial orthopaedic care visits (immobilisation, radiographs) are complete, so patient-reported outcomes have been temporally standardised and are unliked from the orthopaedic visits themselves.

Distal radius fractures are common and treatment strategies are widely divergent. This randomised clinical trial has significant potential to inform clinical practice by comparing the effectiveness of reduction under sedation/anaesthesia to simple immobilisation.

## Supplementary material

10.1136/bmjopen-2024-088273 online supplemental file 1

10.1136/bmjopen-2024-088273 online supplemental file 2

10.1136/bmjopen-2024-088273 online supplemental file 3

## References

[1] Brudvik C, Hove LM (2003). Childhood fractures in Bergen, Norway: identifying high-risk groups and activities. J Pediatr Orthop.

[2] Cooper C, Dennison EM, Leufkens HG (2004). Epidemiology of Childhood Fractures in Britain: A Study Using the General Practice Research Database. J Bone Miner Res.

[3] Naranje SM, Erali RA, Warner WC (2016). Epidemiology of Pediatric Fractures Presenting to Emergency Departments in the United States. J Pediatr Orthop.

[4] Rockwood CA, Beaty JH, Kasser JR (2010). Rockwood and Wilkins’ fractures in children.

[5] Bae DS, Howard AW (2012). Distal radius fractures: what is the evidence?. J Pediatr Orthop.

[6] Crawford SN, Lee LSK, Izuka BH (2012). Closed treatment of overriding distal radial fractures without reduction in children. J Bone Joint Surg Am.

[7] Price CT, Scott DS, Kurzner ME (1990). Malunited forearm fractures in children. J Pediatr Orthop.

[8] Georgiadis AG, Burgess JK, Truong WH (2020). Displaced Distal Radius Fracture Treatment: A Survey of POSNA Membership. J Pediatr Orthop.

[9] Gibbons CL, Woods DA, Pailthorpe C (1994). The management of isolated distal radius fractures in children. J Pediatr Orthop.

[10] McLauchlan GJ, Cowan B, Annan IH (2002). Management of completely displaced metaphyseal fractures of the distal radius in children. A prospective, randomised controlled trial. J Bone Joint Surg Br.

[11] Bernthal NM, Mitchell S, Bales JG (2015). Variation in practice habits in the treatment of pediatric distal radius fractures. J Pediatr Orthop B.

[12] Colaris JW, Allema JH, Biter LU (2013). Re-displacement of stable distal both-bone forearm fractures in children: a randomised controlled multicentre trial. Injury.

[13] Seiler M, Heinz P, Callegari A (2021). Short and long-arm fiberglass cast immobilization for displaced distal forearm fractures in children: a randomized controlled trial. Int Orthop.

[14] Adrian M, Wachtlin D, Kronfeld K (2015). A comparison of intervention and conservative treatment for angulated fractures of the distal forearm in children (AFIC): study protocol for a randomized controlled trial. Trials.

[15] Bohm ER, Bubbar VIC, Hing KY (2006). ABOVE AND BELOW-THE-ELBOW PLASTER CASTS FOR DISTAL FOREARM FRACTURES IN CHILDREN. J Bone Joint Surg Am.

[16] Joeris A, Lutz N, Blumenthal A (2017). The AO Pediatric Comprehensive Classification of Long Bone Fractures (PCCF). Acta Orthop.

[17] Zhao W, Hill MD, Palesch Y (2015). Minimal sufficient balance-a new strategy to balance baseline covariates and preserve randomness of treatment allocation. Stat Methods Med Res.

[18] Gerull WD, Okoroafor UC, Guattery J (2020). Performance of Pediatric PROMIS CATs in Children With Upper Extremity Fractures. *Hand (N Y*).

[19] Quinn H, Thissen D, Liu Y (2014). Using item response theory to enrich and expand the PROMIS® pediatric self report banks. Health Qual Life Outcomes.

[20] Hudak PL, Amadio PC, Bombardier C (1996). Development of an upper extremity outcome measure: the DASH (disabilities of the arm, shoulder and hand) [corrected]. The Upper Extremity Collaborative Group (UECG). Am J Ind Med.

[21] Wong-Baker FACES Foundation (2020). Wong-baker faces pain rating scale.

[22] Forrest CB, Bevans KB, Pratiwadi R (2014). Development of the PROMIS ® pediatric global health (PGH-7) measure. Quality of life research: an international journal of quality of life aspects of treatment, care and rehabilitation. Qual Life Res.

[23] Forrest CB, Tucker CA, Ravens-Sieberer U (2016). Concurrent validity of the PROMIS pediatric global health measure. Quality of life research: an international journal of quality of life aspects of treatment, care and rehabilitation. Qual Life Res.

