# Supplementary material for: Distal Radius Interventions for Fracture Treatment (DRIFT) trial: study protocol for a multicentre randomised clinical trial of completely translated distal radius fractures at paediatric hospitals in North America
Source: BMJ Open. 2025 Oct 29;15(10):e088273. doi: 10.1136/bmjopen-2024-088273 (PMC12574372; doi:10.1136/bmjopen-2024-088273)
Supplement: online supplemental file 1 [file bmjopen-15-10-s001.docx]

**ASSENT FORM**

**For children ages 7 – 10 years of age**

| **Sponsor / Study Title:** | **National Institutes of Health / “DRIFT Trial - Distal Radius Interventions for Fracture Treatment”** |
| --- | --- |
| **Protocol Number:** | **Pro00062090** |
| **Principal Investigator:**  **(Study Doctor)** | **«PiFullName»** |
| **Telephone:** | **«IcfPhoneNumber»** |
| **Address:** | **«PiLocations»** |

Name of participant Age

If you have any questions about what is written below please ask them.

1. **Why are you doing this?**

I would like to tell you about a research study and see if you would like to be in it. Please ask me, other study staff, or your parent to explain any words you don’t understand about the study.

Research studies help us find better ways to take care and treat children who are sick or hurt.

This study is trying to find out the best way to treat children who have broken their arm. Half the children will be treated in a cast, allowing their arm to straighten out on its own, and half will have their broken bone treated with a procedure to straighten the arm. Both of these are used by doctors to treat broken bones.

You do not have to be in the study if you do not want to. You can also decide to start the study now and then stop being in it at any time. No one will be mad at you if you decide not to be in the study or decide to stop being in it later.

1. **What will I do and how long will it take?**

If you want to be in the study, this is what will happen:

Other children will be enrolled in this study. If you want to be in this study, you will be put into one of two groups: procedure or casting.

You will be chosen to be in a group by chance, like flipping a coin.

Procedure: You will be given medicine to make you relax or sleep. Then, the bone in your arm will be straightened out and put in a cast. The procedure is not different from how the doctor would treat a broken arm.

Casting: If you are put into this group, you will get some pain medication and your arm will be put in a cast in the Emergency Room (ER) or the clinic. If your arm was put in a cast before in the ER, no other things will happen.

You will come to the doctor’s office for follow-up visits within 3-14 days of when you were hurt. The doctor will check your arm.

There will be other visits. During these doctor visits, regular x-rays (pictures of your bones) will be taken to make sure that your arm is healing well.

You will also help your parents answer questions about your arm and how you are doing at these visits. Your parents will be sent other surveys by email or text.

Surveys should only take a few minutes to complete and will ask you questions about how your arm is doing. You will not be asked to return to clinic for these surveys.

1. **Do I have to be in this research study and can I stop if I want to?**

You don’t have to take part in this study if you don’t want to. No one will get angry or upset if you do not want to be in the study. Just tell us. You can change your mind later if you decide you don’t want to be in the study anymore.

1. **Could it make me sick [or sicker]?**

Sometimes things happen to people in research studies that may hurt them or make them feel bad. These are called risks. Risks do not happen very often.

All children with broken bones may also have hurt nerves or veins or swelling which can cause injury to muscles.

The risks of the cast include:

- pain and or no feeling because of swelling
- heat injury, your skin gets hot under the cast
- sores from rubbing of the cast, which could get infected
- sore wrist
- stiffness
- hurt nerves around the wrist
- position of the bones may change and you may need to have a surgery or recasted

Risks of the medicine to make you sleep in the Emergency Department or operating room could give you breathing problems but not in a healthy person.

There is also the risk that your bones may not heal correctly.

Having x-rays (pictures of your broken bone) and risks of seeing the pictures of your broken bone which may make you feel uncomfortable.

1. **Will anyone know that I am in this research study?**

What you tell the study doctor or anything else about you may be written down. What is written down about you will be seen by the study doctor, and other people who run and manage the study.

People who make sure that the study is being done the right way may also see it. If the information about the study is sent anywhere else, it will not have your name on it.

1. **How will this research help me or other people?**

There may be a benefit to you from participating in the study since you will see the doctor more often.

We do not know which treatment is better or if there is any difference between them so you may, by chance, have a better outcome from the study.

Other children who have broken arms may be helped in the future.

1. **Can I do something else instead of this research?**

You can decide not to be in this study.

If you decide not to be in this study, your doctor will decide on your treatment which may include one of the two treatments you would have gotten by chance if you were in this study.

1. **Who do I talk to if I have questions?**

You can ask questions at any time. You can ask now. You can ask later.

You can talk to me or you can talk to someone else at any time during the study. Your parents can also contact the study doctor at the number listed on the first page of this form.

🞏 By checking this box, the participant is unable to sign their full signature and verbally agrees to participate in this study

Signature of Child Participant Date

Assent obtained by:

_____________________________________

Signature of Person Obtaining Assent Date

_____________________________________

Printed Name of Person Obtaining Assent
