# Supplementary material for: Distal Radius Interventions for Fracture Treatment (DRIFT) trial: study protocol for a multicentre randomised clinical trial of completely translated distal radius fractures at paediatric hospitals in North America
Source: BMJ Open. 2025 Oct 29;15(10):e088273. doi: 10.1136/bmjopen-2024-088273 (PMC12574372; doi:10.1136/bmjopen-2024-088273)
Supplement: online supplemental file 2 [file bmjopen-15-10-s002.docx]

# **INFORMED CONSENT FORM**

**Parent/Legal Guardian**

| **Sponsor / Study Title:** | **National Institutes of Health / “DRIFT Trial - Distal Radius Interventions for Fracture Treatment”** |
| --- | --- |
| **Protocol Number:** | **Pro00062090** |
| **Principal Investigator:**  **(Study Doctor)** | **«PiFullName»** |
| **Telephone:** | **«IcfPhoneNumber»** |
| **Address:** | **«PiLocations»** |

## Key information

There are different ways to treat distal radius fractures (one of the two long bones in the forearm — breaks close to the wrist) but doctors do not know which treatment is better. This research study is being done to compare two common treatments that are used by orthopedic surgeons today to see which one is more effective (works better). Participants will be randomly assigned to a treatment group (like the flip of a coin). These treatments are:

- Reduction with sedation: The bone will be put back into position (straightened out) and the wrist will be casted while your child is given general anesthesia or sedation. Sometimes a metal plate or wires are used to keep the bone from moving again.
- Simple immobilization: Your child’s wrist will be casted without sedation and the bone will be allowed to heal on its own.

You and your child will also be asked to complete satisfaction, pain, and quality-of-life surveys at scheduled time points. The study will last for 3 years after your child’s study treatment.

The greatest risks of this study are the risks related to procedures for fracture treatment. These include complications associated with anesthesia or sedation with reduction (moving the bone back to alignment). Your child’s surgeon will explain these risks to you.

There may be a benefit to your child from participating in the study since your child will be monitored more actively and for a longer time by the study team than would be typical with this injury. We do not know which study treatment is better or if there is any difference between the study treatments. Hence, your child may, by chance, have a better outcome from the study. This study will help study doctors learn more about treatment for distal radius fractures and it is hoped that this information will help in the treatment of future patients with conditions like your child’s.

You do not have to allow your child to participate in this research study. If you choose for them not to be in this study, the treatment your child receives will be determined by their surgeon and you. Treatment options will likely include the two treatments in the study.

Please read this form carefully. Take your time to ask the study doctor or study staff as many questions about the study as you would like. The study doctor or study staff can explain words or information that you do not understand. Reading this form and talking to the study doctor or study staff may help you decide whether to take part or not. If you decide to take part in this study, you must sign your name at the end of this form and date it.

**Background and purpose**

Your child is being asked to take part in this research study because they have a displaced distal radius fracture. A distal radius fracture is a broken bone at the end of the radius bone near the wrist. This fracture is one of the most common types of broken bones.

Children ages 4 to 10 years old are being asked to take part in this study. About 334 participants will be enrolled in this research study across all study hospitals. Many doctors and children’s hospitals across North America are enrolling participants into this study.

**What will happen during this study?**

Whether or not your child participates in this study, the fracture will be prevented from moving (by a splint or a cast) in the emergency room or orthopedic clinic. Your child will be given pain medicine to feel comfortable.

If you decide to participate, your child will be randomly assigned to one of two study treatment groups. This means that whichever study treatment your child will get will be decided by chance, like flipping a coin. Half the children will be treated in a cast that we call simple immobilization, allowing their arm to straighten out on its own, and half of the children will have their broken bone treated with a procedure under sedation or anesthesia to straighten the arm.

The two common treatments that will be compared in this study are:

- Reduction with sedation: The alignment of the bone will be restored (straightened out which is called “reduction”) and the wrist will be casted while your child is sedated. Sometimes the study doctors may have to make a cut in the arm to insert plates or wires to hold the bones in position, which would need to be removed at a later point.
- Simple immobilization: Your child’s wrist will be casted without sedation.

Your child will be treated the same way in follow-up for their fracture no matter which study treatment they receive and whether or not they participate in the study. Children in both study treatment groups, as well as those who chose not to participate in the study, will be seen regularly to monitor healing of the fracture. All children will follow-up in the orthopedic surgeon’s office according to the surgeon’s standard of care. A return for the first follow-up visit will occur within 3-14 days of study treatment at which time the wrist will be examined. Medications or treatments/procedures are determined by clinical need. We will also collect medical record data including x-ray images from the children taking part in this study, any complications associated with study treatment, and whether healing remains appropriate.

**Expectations**

If your child is enrolled in the study, you will be asked to complete several short surveys over the course of the study regarding how your child is doing. Surveys are to be completed electronically and will be sent to you in the method that you prefer. These surveys will ask questions about how well your child can use their wrist, how much pain they are experiencing, how satisfied you and your child are with treatment, and how much treatment or the injury has affected your or your child’s life and overall health.

Your child’s participation in this study will last approximately 3 years after treatment with study participation from 3 months onwards consisting of electronic surveys

**Risks, side effects, and/or discomforts**

Patients with distal radius fractures are generally at minimal risk of problems if treated appropriately.

For children in the arm of the study that will receive “conscious sedation”:

The risks of sedation include:

- An allergic reaction,
- Aspiration (fluid going into the lungs),
- Over-sedation,
- Mouth or throat pain,
- Hoarseness,
- Injury to mouth or teeth,
- Nausea and vomiting,
- In addition, the IV used may cause a bruise. Occasionally, an infection develops at the IV site.

Uncommonly, your doctor may determine that an operation is needed after the “conscious sedation” procedure. In this case, a child may go to the operating room and have a “formal” procedure, and implants may left inside the body to hold the bone. This will be done under general anesthesia. You should be aware that the risks of general anesthesia include:

- Nausea
- Vomiting
- Blood vessel injury
- Nerve injury
- Lung injury
- Heart attack
- Allergy to drugs
- Brain damage
- Death

Risks of operative treatment of the fracture include:

- Infection due to bacteria that enter the wound
- Pain
- Injury to the nerves or arteries
- Potential growth plate injury
- Alignment problems
- Poor bone healing

These risks are very rare in a healthy child. The surgeon for your child’s treatment will also explain the risks related to sedation separately.

Risks of cast or splint immobilization include:

- Compartment syndrome (pain and/or numbness due to swelling)
- Heat injury (skin burn under cast)
- Pressure sores and skin breakdown
- Infection
- Joint stiffness
- Local nerve damage

The cast or splint will be applied by a health care provider with standard pain medications to control your child’s pain, consistent with any procedure on an awake child. Other optional medicines might include oral medications, anxiolytics, hematoma blocks, or other medications.

It is unknown if your child will experience more discomfort with either study treatment in the early weeks after injury.

In either treatment, bones may move within the cast and require further treatment.

X-rays: Treatment for distal radius fractures includes getting x-rays. One of the possible risks of having x-rays includes cancer due to cumulative exposure to radiation. The amount of radiation from the multiple x-rays that your child will receive is a low risk and is within the range that surgeons think is acceptable for treatment of broken bones. Participants will be shielded as much as possible to prevent unnecessary exposure. X-rays are part of the routine assessment of distal radius fractures and therefore a normal part of treatment of your child’s condition.

**New findings**

Any new important information that is discovered during the study and which may influence your willingness to continue participation in the study will be provided to you.

**Benefits**

To the child:

There may be a benefit to you or your child from participating in the study since your child will be monitored more carefully by the study team. We do not know which study treatment is better or if there is any difference between them so your child may, by chance, have a better outcome from the study. This study will help doctors learn more about treatment for distal radius fractures and it is hoped that this information will help in the treatment of future patients with conditions like your child’s.

To Society:

Most children with these injuries are treated with sedation, but this may be unnecessary for some of them. By being in this study, your child will help researchers learn more about how to best treat displaced distal radius fractures.

**Compensation for participation**

Your child will be paid $40 following each completed survey in the form of an electronic gift card for each of 5 surveys completed for a total of $200 across the study time period.

If your child does not complete the study, for any reason, your child will be paid for each survey they do complete.

If you have any questions regarding your compensation for participation, please contact the study staff.

**Confidentiality**

Records of your child’s participation in this study will be held confidential except when sharing the information is required by law or as described in this informed consent. The study doctor, the sponsor or persons working on behalf of the sponsor, and under certain circumstances, the Institutional Review Board (IRB) will be able to inspect and copy confidential study-related records which identify your child by name. This means that absolute confidentiality cannot be guaranteed. If the results of this study are published or presented at meetings, your child will not be identified.

This study may have some support from the National Institutes of Health (NIH). If so, your child’s study information is protected by a Certificate of Confidentiality. This Certificate allows us, in some cases, to refuse to give out your child’s information even if requested using legal means.

It does not protect information that we have to report by law, such as child abuse or some infectious diseases. The Certificate does not prevent us from disclosing your child’s information if we learn of possible harm to your child or others, or if your child needs medical help.

Disclosures that you consent to in this document are not protected. This includes putting research data in the medical record or sharing research data for this study or future research. Disclosures that you make yourself are also not protected.

A description of this clinical trial will be available on http://www.ClinicalTrials.gov, as required by U.S. Law. This Web site will not include information that can identify you. At most, the Web site will include a summary of the results. You can search this Web site at any time.

To make sure that the health information collected in this study is accurate, it will need to be checked from time to time against your child’s medical records. Some persons may need to see these records (medical information and x-rays) in order to monitor the research and verify the accuracy of the study data, including:

- a limited number of representatives from the study sponsor, National Institutes of Health (namely its monitors and auditors),
- Duke Clinical Research Institute (DCRI). All study data is managed by the Duke Clinical Research Institute,
- the research ethics review board – Advarra IRB (an independent ethics committee that reviewed the ethical aspects of this study to help protect the rights and welfare of study participants),
- your child’s other providers and their staff directly involved in your child’s care, if your child’s provider is a part of your institution’s electronic health information exchange,

Your child’s study records including confidential information about your child collected during the study will be kept at a secure location.

While every effort will be made to protect the privacy of your child’s information, absolute confidentiality cannot be guaranteed. This does not limit the duty of the researchers and others to protect your child’s privacy.

By signing and dating this information and consent form, you consent to the collection, access, use and disclosure of your child’s information as described above.

**Compensation for injury**

If your child becomes ill or is injured while they are in the study, get the medical care that they need right away. You should inform your child’s healthcare professional treating them that they are participating in this study. If you tell the study staff that you think they have been injured, then they will help you get the care you need. You or your insurer may be billed for such treatment.

**Costs**

The costs for standard-of-care treatment for your child’s fracture (reduction with sedation or simple immobilization) will be your responsibility. There are no added costs to you or your child for taking part in this study.

**Future research studies**

Identifiers might be removed from your child’s identifiable private information collected during this study and **could then be used for future research studies or distributed to another study doctor for future research studies** without additional informed consent.

**Study results**

The results of the study will be made available to all participants upon completion of the entire study.

**Whom to contact about the study**

During the study, if your child experiences any medical problems, suffers a research-related injury, or has questions, concerns or complaints about the study such as:

- Whom to contact in the case of a research-related injury or illness;
- Payment or compensation for being in the study;
- You/your child’s responsibilities as a research participant;
- Eligibility to participate in the study;
- The study doctor’s or study site’s decision to exclude your child from participation;
- Results of tests and/or procedures;

**Please contact the study doctor at the telephone number listed on the first page of this consent document.**

If you seek emergency care, or hospitalization is required, alert the treating physician that your child is participating in this research study.

An institutional review board (IRB) is an independent committee established to help protect the rights of research participants. If you have any questions about your child’s rights as a research participant, contact:

- By **mail**:

Study Subject Adviser

Advarra IRB

6100 Merriweather Dr., Suite 600

Columbia, MD 21044

- or call **toll free**:    877-992-4724
- or by **email**:          [adviser@advarra.com](mailto:adviser@advarra.com)

Please reference the following number when contacting the Study Subject Adviser: Pro00062090.

**Voluntary participation/withdrawal**

Your decision to allow your child to participate in this study is voluntary. You may choose to not to allow them to participate or you may withdraw them from the study for any reason without penalty or loss of benefits to which you are otherwise entitled and without any effect on your child’s future medical care. However, please note that any information collected up to the point of your child’s withdrawal cannot be removed from the study.

The study doctor or the sponsor can stop your child’s participation at any time without your consent for the following reasons:

- If it appears to be medically harmful to your child;
- If your child fails to follow directions for participating in the study;
- If it is discovered that your child does not meet the study requirements;
- If the study is canceled; or
- For administrative reasons.

If you decide for your child to stop being part of the study, you should tell your child’s study doctor. Deciding for your child to not be part of the study will not change your child’s regular medical care in any way. No additional data will be collected once you decide to no longer participate in the study.

**Consent**

I have read and understand the information in this informed consent document. I have had an opportunity to ask questions and all of my questions have been answered to my satisfaction. I voluntarily agree to allow my child to participate in this study until I decide otherwise. I do not give up any of my/my child’s legal rights by signing and dating this consent document. I will receive a copy of this signed and dated consent document.

Printed Name of Participant

_____________________________________

Signature of Parent/Legal Guardian Date

Printed Name of Parent/Legal Guardian

Consent obtained by:

_____________________________________

Signature of Person Obtaining Consent Date

_____________________________________

Printed Name of Person Obtaining Consent

**Authorization to Use and Disclose Protected Health Information**

If you decide to allow your child to be in this study, the study doctor and study staff will use and share health data about your child to conduct the study. Health data may include:

- Your name.
- Your child’s name.
- Address.
- Phone number.
- Date of birth.
- Medical history.
- Information from your child’s study visits, including all test results.

Health data may come from your child’s study records or from existing records kept by your child’s doctor or other health care workers.

For this study, the study staff may share health data about your child with authorized users. Authorized users may include:

- Representatives of the National Institutes of Health (NIH).
- Representatives of Advarra IRB (an Institutional Review Board that reviews this study).
- Other research doctors and medical centers participating in this study, if applicable.
- A data safety monitoring board which oversees this study, if applicable.
- Duke Clinical Research Institute (DCRI). All study data is managed by the Duke Clinical Research Institute.

Your child’s health data will be used to conduct and oversee the research.

Once your child’s health data has been shared with authorized users, it may no longer be protected by federal privacy law and could possibly be used or disclosed in ways other than those listed here.

Your permission to use and share health data about your child will end in 50 years unless you revoke it (take it back) sooner.

You may revoke (take back) your permission to use and share health data about your child at any time by writing to the study doctor at the address listed on the first page of this form. If you do this, you will not be able to stay in this study. No new health data that identifies you will be gathered after your written request is received. However, health data about you that has already been gathered may still be used and given to others as described in this form.

Your right to access your child’s health data in the study records will be suspended during the study to keep from changing the study results. When the study is over, you can access your child’s study health data.

If you decide not to sign and date this form, your child will not be able to take part in the study.

**Statement of Authorization**

I have read this form and its contents were explained. My questions have been answered. I voluntarily agree to allow study staff to collect, use and share my child’s health data as specified in this form. I will receive a signed and dated copy of this form for my records. I am not giving up any of my or my child’s legal rights by signing and dating this form.

__________________________________ __________

Signature of Parent/Legal Guardian Date
