# Supplementary material for: Distal Radius Interventions for Fracture Treatment (DRIFT) trial: study protocol for a multicentre randomised clinical trial of completely translated distal radius fractures at paediatric hospitals in North America
Source: BMJ Open. 2025 Oct 29;15(10):e088273. doi: 10.1136/bmjopen-2024-088273 (PMC12574372; doi:10.1136/bmjopen-2024-088273)
Supplement: online supplemental file 3 [file bmjopen-15-10-s003.docx]

| Primary registry and trial-identifying number | ClinicalTrials.gov NCT05131685 |
| --- | --- |
| Date of registration in primary registry | November 23, 2021 |
| Secondary identifying numbers | U01AR079113 |
| Source of monetary or material support | National Institute of Arthritis and Musculoskeletal and Skin Disease (NIAMS) and the National Institute of Child Health and Human Development (NICHD)  The funder is involved in monitoring the ongoing progress of the trial. They will not have any role in the execution, analysis, interpretation of data, or reporting of results. |
| Primary sponsor | Ann & Robert H Lurie Children’s Hospital of Chicago  DRIFT is an investigator-initiated trial. The sponsor is directly involved with the initiation, design, and execution of the trial. |
| Contact for public queries | Jamie Burgess and Candace Young |
| Contact for scientific queries | Jamie Burgess and Candace Young |
| Public Title | Distal Radius Interventions for Fracture Treatment (DRIFT) |
| Scientific Title | Distal Radius Interventions for Fracture Treatment (DRIFT) |
| Countries of recruitment | United States and Canada |
| Health condition(s) or problem(s) studied | Distal radius fractures |
| Interventions | Reduction under sedation – closed reduction under conscious sedation following casting  Simple immobilization – immobilization in cast without reduction |
| Key inclusion and exclusion criteria | Inclusion:   - Males and females, aged 4-10 years inclusive - Diagnosis of 100% dorsally translated metaphyseal fractures of the radius with any or no distal ulna involvement near the same level in the ipsilateral arm - Fracture is acute and less than 5cm from the distal radial growth plate   Exclusion:   - Physeal involvement of fractures - Presence of fractures other than the ulna - Presence of pathologic fracture or open fracture - Metabolic or neuromuscular diagnosis, or bone disease |
| Study type | Multicenter, parallel, open-label, randomized controlled trial  Participants randomized via minimal sufficient balance algorithm |
| Date of first enrollment | April 9, 2023 |
| Target sample size | 334 |
| Recruitment status | Enrolling participants |
| Primary outcome | PROMIS Pediatric CAT – Upper Extremity measured at one year |
| Key secondary outcomes | DASH – S/PA  PROMIS Pediatric Pain Interference  Wong-Baker FACES Pain score  PROMIS Pediatric Global Health – Global Score  PROMIS Pediatric Global Health – Fatigue interference  PROMIS Pediatric Global Health – Pain interference  Number of revisions, refractures, reductions, and reoperations  Other complications  Missed work or school  Satisfaction questionnaire  Cosmesis  Radiographic alignment |
