# Supplementary material for: Distal Radius Interventions for Fracture Treatment (DRIFT) trial: study protocol for a multicentre randomised clinical trial of completely translated distal radius fractures at paediatric hospitals in North America
Source: BMJ Open. 2025 Oct 29;15(10):e088273. doi: 10.1136/bmjopen-2024-088273 (PMC12574372; doi:10.1136/bmjopen-2024-088273)
Supplement: Supplementary file 4 [file bmjopen-2024-088273.reviewer_comments.pdf]

## PEER REVIEW HISTORY

BMJ Open publishes all reviews undertaken for accepted manuscripts. Reviewers are asked to complete a checklist review form and are provided with free text boxes to elaborate on their assessment. These free text comments are reproduced below.

### ARTICLE DETAILS

**Title (Provisional)**

Distal Radius Interventions for Fracture Treatment (DRIFT) trial: Study protocol for a multi-center randomized clinical trial of completely translated distal radius fractures at pediatric hospitals in North America

**Authors**

Balmert Bonner, Lauren; Janicki, Joseph; Georgiadis, Andrew; Truong, Walter; Harris Beauvais, Dorothy; Belthur, Mohan; Daley, Erika L.; Franzone, Jeanne; Howard, Andrew; May, Collin; Rockhold, Frank; Schulz, Jacob; Bailey, Mary; Chiswell, Karen; DeLaRosa, Jesse; Brooks, Jaysson T.; Cantanzano, Anthony A.; Chan, Andrea; Chu, Alice; Dodwell, Emily R; El-Hawary, Ron; Ellis, Henry; Fitzgerald, Ryan; Frick, Steven; Ganley, Theodore J.; Gargiulo, Dominic; Gauthier, Luke; Gill, Corey S.; Goldstein, Rachel; Halsey, Matthew F.; Hardesty, Christina; Ho, Christine; Kaushal, Neil; Lawrence, John Todd; Lee, R. Jay; Leitch, Khristinn K.; Masrouha, Karim; Mitchell, Stuart; OMalley, Natasha; Payares- Lizano, Monica; Perry, Daniel; Ramalingam, Wendy; Rhodes, Jason; Sanders, Julia; Shah, Apurva S.; Sharkey, Melinda; Silva, Mauricio; Silva, Selina; Thompson, Rachael; Vorhies, John; Wright, James Gardner; Young, Candace; Burgess, Jamie

---

### VERSION 1 - REVIEW

---

| | |
|---|---|
| **Reviewer** | **1** |
| **Name** | **Dresing, Klaus** |
| **Affiliation** | **University of Göttingen Institute of General Medicine, Orthopaedics and Plastic Surgery** |
| **Date** | **10-Jul-2024** |
| **COI** | **none** |

---

100% dorsally displaced fractures less than 5cm from the distal radial physis will be recruited for the study.

This inclusive criteria is not exact specified.

Please specify according to the PCCF which group you include. Do you only include metaphyseal fractures (The metaphysis is determined by a square the sides of which have the same length as the widest part of the growth plate):

23-M/3.1, 3.2

23rM/3.1, 23rM/3.2

or

are distal epiphyseal fractures included or excluded.

---

| | |
|---|---|
| **Reviewer** | **2** |
| **Name** | **Schmidt, Viktor** |
| **Affiliation** | **Umea Universitet** |
| **Date** | **14-Jul-2024** |
| **COI** | **None** |

---

Page 11

• Add "Data Safety Monitoring Boards" in parentheses after "DSMB."

Page 13

• The handling of crossover cases remains unclear: Will crossover cases be analyzed based on the original arm they were intended for, or the treatment to which they crossed over? If patients are included in their allocated arm, will this not skew the results? Please clarify.

Page 19

• Consider rephrasing ”100% dorsal displacement” as ”100% dorsal translation” or "ad latus displacement" to avoid confusion with dorsal angulation.

• The inclusion criteria mention ”any ulna involvement”, while the exclusion criteria state ”presence of fractures other than the ulna near the same level in the ipsilateral arm.” This inconsistency may lead to confusion and inclusion of patients with olecranon fractures or similar conditions. Please clarify.

• For the criterion ”fracture is less than 5 cm from the distal radial growth plate”, will any calibration be used for these measurements? Additionally, could this result in classifying diaphyseal fractures in 4-year-olds and meta/diaphyseal fractures in 10-year-olds as the same? Please provide further explanation.

General Questions

• How will you document patients who declined participation and those who were not asked to participate?

• The document does not discuss any limitations. Do you consider there to be no limitations?

• In the methods it is not clear to me whether patients in the reduction treatment arm are evaluated for re-displacement with the possibility for re-reduction at later times or if they should be reduced 1 time only and then evaluated for results. How strict is the protocol on this and is it reasonable to believe that all 34(!) hospitals follow the same method when it is not clearly stated in the method.

---

| | |
|---|---|
| **Reviewer** | **3** |
| **Name** | **Launonen, Antti** |
| **Affiliation** | **Tampere University Hospital** |
| **Date** | **18-Jul-2024** |
| **COI** | **None** |

---

The specific comments are on a separate file. Good work!

Review report of the protocol: *"Distal Radius Interventions for Fracture treatment (DRIFT) trial: Study protocol for a multicenter randomized clinical trial"*

The study is an ambiguous multi-site trial about the children's distal radius fractures. Running this wide setting is complicated and needs lots of energy to push it through. However, the object is important and needed, and without this, a large set-up is needed to achieve the goal. I wish you all the best in this work!

In general, the protocol is well written. Some inconsistencies are present, which I have pointed out.

First, I will address some general comments and, later, the specific comments.

The method of immobilisation wasn't described, please add to the methods.

With this calibre of a large trial, I am sure the target journal will be one of the top orthopaedic journals. I am certain they will require a separate statistical analysis plan. Please consider writing it.

It wasn't clear if the protocol is written according to some prespecified checklist and also if the intention is to follow, e.g. CONSORT, with the final report. Please add the ClinicalTrials handle.

Inconsistencies with abbreviations made it hard to understand who and what was doing which task. Please check. Also, it wasn't very clear who are the main supervisors; at least, the primary investigator(s) would be nice to be named intext.

Lastly, there are quite a few questionnaires. While the patients are small children, is the number of questionnaires overwhelming?

Some specific remarks:

Firstly, the trial's abbreviation collides with the trial I am an author of. The name hasn't been published in our trial but is used as a working name during the trial run-time. The main analysis and report will be published in 2026, and we plan to use this abbreviation (DRIFT) in the title. Please consider if you want to change.

*Hevonkorpi TP, Launonen AP, Raittio L, Luokkala T, Kukkonen J, Reito A, et al. Nordic Innovative Trial to Evaluate OsteoPorotic Fractures (NITEP-group): non-operative treatment versus surgery with volar locking plate in the treatment of distal radius fracture in patients aged 65 and over - a study protocol for a prospective, randomized controlled trial. BMC Musculoskelet Disord. 2018;19(1):106.*

Secondly, since the trial includes children, add the age limits to the title. Now, it is quite general, and the reader might think it's about adults or older adults.

P4L17 "100% dorsally displaced". I am not sure what that means. Without contact among the fracture parts?

P4L21 Those *patients* with…?

P4L37 …and secondary operations?

P5L18 Clinical Coordinating Center?

P6L22 Recently? Depends on the scale…

P8L18 IMPACCT is the consortium formed for this trial or was it formed for other purposes and now used for the trial? You could use it on the title as well.

P8L30 The informational video could be supplementing information or uploaded to the public domain.

P8L32 CCC is an "institution." Please specify who the family will be talking to. You could also elaborate in the previous chapter about the structure of the CCC.

P8L42 Please elaborate the e-consent. At least in the EU, one has to have a strong identification for the proper consent in an electronic environment or give a signature. Also,

as the minor patient is not eligible to give the proper juridical consent, please specify if the "family consent" means one parent or both parents. How will consent be obtained if either legal guardian is absent (grandparents, etc..).

P9L38 How the cross-over patients will be analyzed? ITT?

P9L59 I am not familiar with this method, but as the author notes: "It is important to point out that, when a covariate in included in the randomization algorithm, one should include that covariate in the primary analysis and to interpret the trial results accordingly." This should be discussed or at least noted in the statistical analysis plan (SAP), should you write it.

P10L19 The manuscript uses DCC and CCC inconsistently, but I assume it should be CCC.

P10L35 Please add reference.

P10L39 am? Arm, I assume.

P11L58 Even if the primary outcome is one year? Again, inconsistency with the abbreviation DCC/CCC.

P11L14 DSMB – what is this?

Please rewrite "interim analysis." It was confusing, and I didn't quite understand it. Also, specify who will be on the steering committee and how you will ascertain that the unblinded statistical team will not communicate with the team analysing the results.

P11L42 I certainly hope the patients in this trial are not missing work… I think child labour is illegal in Western countries ☺.

P11L28 CAT—this abbreviation hasn't been used before. Also, please elaborate on the PROMIS usage and validation. The patients are children, so how is it with kids to obtain a valid response?

P14L38 and additional visits/difference between the arms to health-care departments?

P15L41 What is R34? Did the participants respond also about the primary outcome?

---

**Reviewer** 4

**Name** Belloti, João

**Affiliation** Federal University of São Paulo (UNIFESP/EPM), Orthopedics and Traumatology - Division of Hand Surgery and Upper Limb

**Date** 23-Jul-2024

**COI** **none**

---

The manuscript is a protocol for a multicenter pragmatic randomized clinical trial for the conservative treatment of completely displaced fractures of the distal end of the radius in children. The interventions used in the study are: fracture reduction under anesthesia and immobilization compared to simple immobilization.

The protocol addresses a frequent topic and its methodological design is relevant, however there are some very relevant points that must be reviewed to consider publishing this manuscript:

- In the manuscript there is no information about the registration data or beginning of inclusion of patients. Checking registration NCT05131685, the study is ongoing, starting on 2022-02-16.

- The title of the study is inadequate, it does not describe the population of interest or what type of distal radius fracture will be studied.

- In the introduction, the authors describe that there is no good quality evidence, based on randomized clinical studies, to prove clinical support for the treatment of these fractures, however there are several RCTs in the literature on this topic 1-5, including the publication of a protocol for a multicenter RCT protocol (Adrian-2015), on the treatment of patients with fractures of the distal extremity of the forearm and metaphyseal fractures of the radius, studies that should be considered.

Interventions

- Although it is a pragmatic RCT, there is a need to describe the possible methods that will be used to carry out the two interventions evaluated. What are the possible types of plaster casts or orthoses that can be used? What will be the fracture reduction methods? Will rehabilitation be carried out?

Outcomes

- Who will carry out the assessments and how will data be collected at 6 weeks and 3 months of follow-up (page 7, line 36)?

- What will be the criteria for fracture reducibility, loss of reduction and possible need for remanipulation or failure of reduction?

- How the secondary outcome Cosmesis will be measured (table 1; page 7, line 12)?

- The radiographic outcome (page 11, line 41) is not included in Table 1. What will be the method and what radiographic parameters will be measured?

- The cost-effectiveness analysis method (page 13, line 3) needs to be clearly described, how and when it will be carried out?

- What outcomes and how will they be measured in the analyzes carried out after one year of follow-up (page 13, line 3)?

1- McLauchlan GJ, Cowan B, Annan IH, Robb JE. Management of completely displaced metaphyseal fractures of the distal radius in children. A prospective, randomised controlled trial. J Bone Joint Surg Br. 2002 Apr;84(3):413-7. doi: 10.1302/0301-620x.84b3.11432.

2- Colaris JW, Allema JH, Biter LU, de Vries MR, van de Ven CP, Bloem RM, Kerver AJ, Reijman M, Verhaar JA. Re-displacement of stable distal both-bone forearm fractures in children: a randomised controlled multicentre trial. Injury. 2013 Apr;44(4):498-503. doi: 10.1016/j.injury.2012.11.001.

3- Seiler M, Heinz P, Callegari A, Dreher T, Staubli G, Aufdenblatten C. Short and long-arm fiberglass cast immobilization for displaced distal forearm fractures in children: a randomized controlled trial. Int Orthop. 2021 Mar;45(3):759-768. doi: 10.1007/s00264-020-04800-w.

4- Adrian M, Wachtlin D, Kronfeld K, Sommerfeldt D, Wessel LM. A comparison of intervention and conservative treatment for angulated fractures of the distal forearm in children (AFIC): study protocol for a randomized controlled trial. Trials. 2015 Sep 30;16:437. doi: 10.1186/s13063-015-0912-x.

5- Bohm ER, Bubbar V, Yong Hing K, Dzus A. Above and below-the-elbow plaster casts for distal forearm fractures in children. A randomized controlled trial. J Bone Joint Surg Am. 2006 Jan;88(1):1-8. doi: 10.2106/JBJS.E.00320.

---

## VERSION 1 - AUTHOR RESPONSE

**Reviewer:** 1
Dr. Klaus Dresing, University of Göttingen Institute of General Medicine
Comments to the Author:

1. *100% dorsally displaced fractures and less than 5cm from the distal radial physis will be recruited for the study. This inclusive criteria is not exact specified.*

   We appreciate attention drawn to this point and will clarify response below, to your second point. The abstract has been amended to include "100% dorsally translated metaphyseal fractures of the radius" (Page 2, L8). This has also been added to the Methods section (Page 6, L22-24) and updated in the Inclusion Criteria list (Page 19, Box 1).

2. *Please specify according to the PCCF which group you include. Do you only include metaphyseal fractures (The metaphysis is determined by a square the sides of which have the same length as the widest part of the growth plate):*
   *23-M/3.1, 3.2*
   *23rM/3.1, 23rM/3.2*
   *or are distal epiphyseal fractures included or excluded.*

Distal epiphyseal fractures are excluded. We recognize the importance of clarity for readers familiar with the AO-PCCF classification, and all fractures will be 23-M/3.1 and 23r-M/3.1, now specified in "Participants" section of the Methods (Page 6, L22-24).

**Reviewer:** 2
Dr. Viktor Schmidt, Umea Universitet
Comments to the Author:

1. *Page 11 Add "Data Safety Monitoring Boards" in parentheses after "DSMB."*

   We apologize for this oversight and have add the full name “Data and Safety Monitoring Board” to the manuscript the first place “DSMB” is referenced (Page 10, L13).

2. *Page 13 The handling of crossover cases remains unclear: Will crossover cases be analyzed based on the original arm they were intended for, or the treatment to which they crossed over? If patients are included in their allocated arm, will this not skew the results? Please clarify.*

   In primary analyses, all participants will be analyzed according to the arm to which they were randomized, regardless of the intervention actually received. This aligns with the intention-to-treat (ITT) principle and allows for an unbiased estimate of the treatment policy effect, preserving the randomization. The ITT analysis is generally the recommended primary analysis in a superiority trial. A subsequent per-protocol analysis is planned to assess the robustness of the ITT results. In this analysis, outcomes from participants who crossed over will only be included up until the time of cross-over (i.e., censored at the time of cross-over). For example, a participant who is randomized to the immobilization arm but later receives reduction will only contribute outcomes from any assessments made prior to the reduction. We have revised this section to provide additional clarity (Page 12, L9-10; Page 13, L1-2).

3. *Page 19 Consider rephrasing ”100% dorsal displacement” as ”100% dorsal translation” or "ad latus displacement" to avoid confusion with dorsal angulation.*

   This has been changed in the inclusion criteria to read “100% dorsally translated metaphyseal fractures of the radius”. (Page 19, Box 1).

4. *The inclusion criteria mention ”any ulna involvement”, while the exclusion criteria state ”presence of fractures other than the ulna near the same level in the ipsilateral arm.” This inconsistency may lead to confusion and inclusion of patients with olecranon fractures or similar conditions. Please clarify.*

   The Inclusion Criteria have been updated to read "distal ulna involvement" for clarity (Page 19, Box 1).

5. *For the criterion ”fracture is less than 5 cm from the distal radial growth plate”, will any calibration be used for these measurements? Additionally, could this result in classifying diaphyseal fractures in 4-year-olds and meta/diaphyseal fractures in 10-year-olds as the same? Please provide further explanation.*

   Thank you for the question. The Authors agree that there is some degree of interpretation involved in the assessment of eligible fractures. No measurement is involved, since there are various definitions of "metaphyseal." Five cm was selected because that approximately the maximal extent of the metaphysis in an average 5 year old (Wu C, Wang D, Mo Y, Zhang Z, Ning B. Characteristics of the length of the radius and ulna in children. Front Pediatr. 2022 Aug 9;10:737823. doi: 10.3389/fped.2022.737823. PMID: 36016874; PMCID:

PMC9395915.) and diaphyseal fractures in younger children are considered to be acceptable candidates for non-reduction treatment in the diaphysis. This is also consistent with the work of Laaksonen (Laaksonen T, Kosola J, Nietosvaara N, Puhakka J, Nietosvaara Y, Stenroos A. Epidemiology, Treatment, and Treatment Quality of Overriding Distal Metaphyseal Radial Fractures in Children and Adolescents. J Bone Joint Surg Am. 2022 Feb 2;104(3):207-214. doi: 10.2106/JBJS.21.00850. PMID: 34780389.) who found that fractures were overwhelmingly concentrated in the distal metaphysis, itself extending about 4.x cm from the physis at the age of 5. To be a practical trial, 5 cm would likely eliminate patients who had true metaphyseal fractures at older ages, and is therefore a conservative threshold for the majority of this group. Also, the inclusion of the word "diaphyseal" also constrains surgeon from including fractures that are clearly diaphyseal, regardless of centimeter distance.

6. *How will you document patients who declined participation and those who were not asked to participate?*

   Families that are not asked to participate will not be documented. Families who are approached but decline participation will be added to a screening log. The screening surveys will include demographic information including age, sex, race, ethnicity, who approached the patient, and- if they decline participation – reason why and what their ultimate treatment was. Sites maintain screening logs to document screen failures and refusals. This has been added into the manuscript in the Participants section (Page 7, Lines 13-17).

7. *The document does not discuss any limitations. Do you consider there to be no limitations?*

   The Authors appreciate attention drawn to this point. A Discussion section has been added to review the implications of the trial and potential limitations of the trial design (Pages 16-17).

8. *In the methods it is not clear to me whether patients in the reduction treatment arm are evaluated for re-displacement with the possibility for re-reduction at later times or if they should be reduced 1 time only and then evaluated for results. How strict is the protocol on this and is it reasonable to believe that all 34(!) hospitals follow the same method when it is not clearly stated in the method.*

   This is an excellent point and is now better clarified in the manuscript text. As this is a pragmatic study, we allow for additional reduction, cast changes, cast wedging per the surgeon's discretion. Even if the patient is given immobilization only at initial presentation, the surgeon and family may decide, after follow up to undergo a closed reduction under anesthesia for unacceptable alignment. All of these scenarios and any other occurrences beyond a single initial casting will be documented and followed in the assigned arm. Sub-analyses will be performed to evaluate the effects of these additional interventions. In the "Interventions" section of the Methods, the following text has been added which continues the repeated theme of a practical trial: " This means that later surgery, subsequent manipulation, or any other care decision is completely unconstrained by the trial and will be recorded." (Page 8, Lines 17-18)

**Reviewer**: 3
Dr. Antti Launonen, Tampere University Hospital
Comments to the Author:
The specific comments are on a separate file. Good work!

*Review report of the protocol: "Distal Radius Interventions for Fracture treatment (DRIFT) trial: Study protocol for a multicenter randomized clinical trial" The study is an ambiguous multi-site trial about the children's distal radius fractures. Running this wide setting is complicated and needs lots of energy to push it through. However, the object is important and needed, and without this, a large set-up is needed to achieve the goal. I wish you all the best in this work! In general, the protocol is well written. Some inconsistencies are present, which I have pointed out. First, I will address some general comments and, later, the specific comments.*

1. *The method of immobilisation wasn't described, please add to the methods.*

   This is a pragmatic trial. Hence, the method of immobilization is per surgeon discretion and will be documented. Immobilization can include long arm cast, short arm cast, or splints per the discretion of the provider. The key difference is whether or not anesthesia or conscious sedation was used to attempt to reduce the forearm to its original length and alignment. Additional clarity has been added to the Interventions section (Page 8, Lines 19-22).

2. *With this calibre of a large trial, I am sure the target journal will be one of the top orthopaedic journals. I am certain they will require a separate statistical analysis plan. Please consider writing it. It wasn't clear if the protocol is written according to some prespecified checklist and also if the intention is to follow, e.g. CONSORT, with the final report. Please add the ClinicalTrials handle.*

   We appreciate this important point. A statistical analysis plan (SAP) was developed during the design phase (V1.0 9/7/2023). We plan to make the SAP available when publishing the final trial results. The ClinicalTrials registration number (NCT05131685) was included below the abstract (Page 2, Line 22). We have added reference to following CONSORT guidelines for reporting results to the Statistical analyses section (Page 13, Lines 6-7).

3. *Inconsistencies with abbreviations made it hard to understand who and what was doing which task. Please check.*

   We apologize for the confusion with abbreviations. We have added a section on the study organization to the Study design section to clarify the roles of the Data Coordinating Center (DCC) and the Clinical Coordinating Center (CCC) (Page 6, Lines 6-15).

4. *Also, it wasn't very clear who are the main supervisors; at least, the primary investigator(s) would be nice to be named intext.*

   We have added reference to the study PI (Dr. Janicki) to the study organization section (as referenced above).

5. *Lastly, there are quite a few questionnaires. While the patients are small children, is the number of questionnaires overwhelming?*

   This is a good point. The patient reported outcome surveys will be completed by parents or caregivers. In particular, the primary outcome (Pediatric-PROMIS-UE) is a computer adaptive test that is available for children age 8-17, but can filled out by parent proxy in the age group of 5-17 years. Children under age 8 will have parent proxy, and all children will have the option of parent proxy. Parent proxy for the primary outcome has been well validated in this age group. A reference has been added regarding validation of the primary endpoint (Page 10, Line 21).

*Some specific remarks:*

6. *Firstly, the trial's abbreviation collides with the trial I am an author of. The name hasn't been published in our trial but is used as a working name during the trial run-time. The main*

*analysis and report will be published in 2026, and we plan to use this abbreviation (DRIFT) in the title. Please consider if you want to change.*
*Hevonkorpi TP, Launonen AP, Raittio L, Luokkala T, Kukkonen J, Reito A, et al. Nordic Innovative*
*Trial to Evaluate OsteoPorotic Fractures (NITEP-group): non-operative treatment versus surgery*
*with volar locking plate in the treatment of distal radius fracture in patients aged 65 and over – a study protocol for a prospective, randomized controlled trial. BMC Musculoskelet Disord. 2018;19(1):106.*

We appreciate your concern of overlap in the trial abbreviation. However, given that we have already registered the trial (ClinicalTrials.gov) and recruitment is ongoing, we seem obligated to continue using the DRIFT abbreviation.

7. *Secondly, since the trial includes children, add the age limits to the title. Now, it is quite general, and the reader might think it's about adults or older adults.*

   We appreciate this comment and have revised title of this protocol manuscript to read Distal Radius Interventions for Fracture Treatment (DRIFT) trial: Study protocol for a multi-center randomized clinical trial of completely translated distal radius fractures in children.

8. *P4L17 "100% dorsally displaced". I am not sure what that means. Without contact among the fracture parts?*

   Another Reviewer also felt this could interpreted in different ways. We have changed the language to read 100% dorsally translated metaphyseal fractures of the radius in several instances in the manuscript.

9. *P4L21 Those patients with…?*

   We have revised this sentence to read "Those patients with open fractures…" (Page 2, Line 9)

10. *P4L37 …and secondary operations?*

    We have revised the text to reference the additional secondary outcome of additional procedures (revisions, refractures, reductions, and reoperations). (Page 2, Lines 17-18)

11. *P5L18 Clinical Coordinating Center?*

    The randomization will occur by the Data Coordinating Center (DCC), who is responsible for data management and statistical analyses. We have added additional clarity to the study design section around the organizational structure (DCC vs. CCC). (Page 6, Lines 6-15).

12. *P6L22 Recently? Depends on the scale…*

    Agree, it does depend. We have removed the word "recently". (Page 4, Line 10)

13. *P8L18 IMPACCT is the consortium formed for this trial or was it formed for other purposes and now used for the trial? You could use it on the title as well.*

    The consortium was formed as a group in which multiple trials could be run, including this one. The Authors think that mention of the study group might be cumbersome and prefer to mention it in the methods and eventual Authorship byline. (Page 6, Lines 20-21).

14. *P8L30 The informational video could be supplementing information or uploaded to the public domain.*

The information video is in the public domain and can be found at drift-study.digitrial.com. The video is now cited in the manuscript text, with a public URL that any reader can visit. (Page 7, Lines 2-3)

15. *P8L32 CCC is an "institution." Please specify who the family will be talking to. You could also elaborate in the previous chapter about the structure of the CCC.*

The CCC is staffed by research personnel. We have clarified this in the manuscript and added a section regarding the organizational structure as mentioned above.

16. *P8L42 Please elaborate the e-consent. At least in the EU, one has to have a strong identification for the proper consent in an electronic environment or give a signature. Also, as the minor patient is not eligible to give the proper juridical consent, please specify if the "family consent" means one parent or both parents. How will consent be obtained if either legal guardian is absent (grandparents, etc..).*
In this study, ethics has agreed that it is low risk since it is comparing two standard of care treatments. Therefore, only one parental consent is required. This has been added to the Participants section of the manuscript. We have also added a description of the assent process for children ages 7-10 years old. (Page 7, Lines 6-12)

17. *P9L38 How the cross-over patients will be analyzed? ITT?*

As primary analyses will be based on the intention-to-treat principle, participants will be analyzed according to their randomized assignment (regardless of what intervention they actually received). A secondary per-protocol analysis will be conducted whereby participants will be included in their randomized group up until the time of treatment cross-over. Follow-up will be censored at the time of treatment cross-over. This description is included in the Analysis populations and estimands section. We have revised for clarity. (Page 12, Lines 9-10; Page 13, Lines 1-2).

18. *P9L59 I am not familiar with this method, but as the author notes: "It is important to point out that, when a covariate in included in the randomization algorithm, one should include that covariate in the primary analysis and to interpret the trial results accordingly." This should be discussed or at least noted in the statistical analysis plan (SAP), should you write it.*

Thank you for this comment, and we completely agree. The Statistical analyses section of the manuscript describes plans to incorporate all covariates included in the randomization algorithm (sex, age, center) in the analysis (Page 13, Lines 11-13). This has also been detailed in the separate SAP.

19. *P10L19 The manuscript uses DCC and CCC inconsistently, but I assume it should be CCC.*

There are two separate entities involved in the organizational structure, the Data Coordinating Center (DCC) and the Clinical Coordinating Center (CCC). We have added additional details regarding each entity in the Study Design section as described above.

20. *P10L35 Please add reference.*

The following reference has been added to the manuscript where the primary outcome is introduced (Sample size considerations) (Page 9, Line 19):

Gerull WD, Okoroafor UC, Guattery J, Goldfarb CA, Wall LB, Calfee RP. Performance of Pediatric PROMIS CATs in Children With Upper Extremity Fractures. Hand (N Y). 2020 Mar;15(2):194-200. doi: 10.1177/1558944718793195. Epub 2018 Aug 6. PMID: 30081662; PMCID: PMC7076605.

21. *P10L39 am? Arm, I assume.*

    We have corrected this typo to state "A sample size of 133 per arm…" (Page 9, Line 20)

22. *P11L58 Even if the primary outcome is one year? Again, inconsistency with the abbreviation DCC/CCC.*

    Given the long follow-up time of the primary outcome (1 year), we have chosen to use the 6-month outcome to inform the sample size re-estimation. This will allow for adequate information and time to implement any changes in the sample size. As the re-estimation is solely based on the observed standard deviation of the outcome, we believe the estimate of the standard deviation at 6 months is likely to be similar to the estimate of the standard deviation at 1 year.

23. *P11L14 DSMB – what is this?*

    We have included the full name for this abbreviation where it is first referenced (Data and Safety Monitoring Board). (Page 10, Line 13).

24. *Please rewrite "interim analysis." It was confusing, and I didn't quite understand it. Also, specify who will be on the steering committee and how you will ascertain that the unblinded statistical team will not communicate with the team analysing the results.*

    The proposed interim analysis for sample size re-estimation is designed to allow for modifications to the planned sample size based on the observed data at the time of the interim analysis (and initial trial design assumptions). In particular, this allows for uncertainty in the original assumption regarding the standard deviation of the primary outcome. Briefly, we are updating the original sample size calculations with a revised estimate of the standard deviation based on the data observed at the time of the interim analysis. If the observed standard deviation is larger than the originally assumed standard deviation, increasing the sample size can increase the likelihood that the trial meets its objective of being adequately powered to detect a clinically meaningful treatment effect. We have revised this section for additional clarity (Page 10 Lines 1-13). We have also added additional information regarding the composition of the steering committee. The DCC also developed a blinding plan that describes the approach for handling data and reports; maintaining confidentiality; delineating access to data, randomization information, and outcomes; and communication within and between blinded/unblinded teams.

25. *P11L42 I certainly hope the patients in this trial are not missing work... I think child labour is illegal in Western countries.*

    We have revised the text to clarify that the outcome for 'missed work days' is based on number of missed days of work for the parent(s). (Page 11, Line 1; Page 14, Line 7)

26. *P11L28 CAT—this abbreviation hasn't been used before. Also, please elaborate on the PROMIS usage and validation. The patients are children, so how is it with kids to obtain a valid response?*

    The full name for this abbreviation is first included in the Sample size considerations section where the primary outcome is first referenced. (Computer Adaptive Test (CAT)) (Page 9,

Lines 17-18). As noted in response to #5 above, Pediatric-PROMIS-UE is a computer adaptive test that is available for children age 8-17, but can filled out by parent proxy in the age group of 5-17 years. We have added the reference to Gerull et al. (2020) in the Outcomes section (Page 10, Line 21). The manuscript highlights a study designed to evaluate the performance of this measure when completed by children and by parent-proxy.

27. *P14L38 and additional visits/difference between the arms to health-care departments?*

    We appreciate this comment; however, additional visits to health-care departments in not being documented in this study.

28. *P15L41 What is R34? Did the participants respond also about the primary outcomes*

    We have clarified that this is an NIH grant mechanism specifically for planning and designing subsequent multi-center clinical trials (Page 15, Line 8). There were no data collected on the primary outcome during the R34 planning grant.

**Reviewer**: 4
Dr. João  Belloti, Federal University of São Paulo (UNIFESP/EPM), Universidade Federal de Sao Paulo Escola Paulista de Medicina
Comments to the Author:

*The manuscript is a protocol for a multicenter pragmatic randomized clinical trial for the conservative treatment of completely displaced fractures of the distal end of the radius in children. The interventions used in the study are: fracture reduction under anesthesia and immobilization compared to simple immobilization. The protocol addresses a frequent topic and its methodological design is relevant, however there are some very relevant points that must be reviewed to consider publishing this manuscript:*

1. *In the manuscript there is no information about the registration data or beginning of inclusion of patients. Checking registration NCT05131685, the study is ongoing, starting on 2022-02-16.*

   We have added a sentence to the Ethics and Dissemination section to clarify when the trial was registered on ClinicalTrials.gov, when enrollment began, and when it is anticipated to be completed. (Page 16, Lines 3-5)

2. *The title of the study is inadequate, it does not describe the population of interest or what type of distal radius fracture will be studied.*

   We have revised the title as described above in response to reviewer 3.

3. *In the introduction, the authors describe that there is no good quality evidence, based on randomized clinical studies, to prove clinical support for the treatment of these fractures, however there are several RCTs in the literature on this topic 1-5, including the publication of a protocol for a multicenter RCT protocol (Adrian-2015), on the treatment of patients with fractures of the distal extremity of the forearm and metaphyseal fractures of the radius, studies that should be considered.*
   *1- McLauchlan GJ, Cowan B, Annan IH, Robb JE. Management of completely displaced metaphyseal fractures of the distal radius in children. A prospective, randomised controlled trial. J Bone Joint Surg Br. 2002 Apr;84(3):413-7. doi: 10.1302/0301-620x.84b3.11432.*
   *2- Colaris JW, Allema JH, Biter LU, de Vries MR, van de Ven CP, Bloem RM, Kerver AJ, Reijman M, Verhaar JA. Re-displacement of stable distal both-bone forearm fractures in children: a randomised controlled multicentre trial. Injury. 2013 Apr;44(4):498-503. doi: 10.1016/j.injury.2012.11.001.*
   *3- Seiler M, Heinz P, Callegari A, Dreher T, Staubli G, Aufdenblatten C. Short and long-arm*

*fiberglass cast immobilization for displaced distal forearm fractures in children: a randomized controlled trial. Int Orthop. 2021 Mar;45(3):759-768. doi: 10.1007/s00264-020-04800-w.*
*4- Adrian M, Wachtlin D, Kronfeld K, Sommerfeldt D, Wessel LM. A comparison of intervention and conservative treatment for angulated fractures of the distal forearm in children (AFIC): study protocol for a randomized controlled trial. Trials. 2015 Sep 30;16:437. doi: 10.1186/s13063-015-0912-x.*
*5- Bohm ER, Bubbar V, Yong Hing K, Dzus A. Above and below-the-elbow plaster casts for distal forearm fractures in children. A randomized controlled trial. J Bone Joint Surg Am. 2006 Jan;88(1):1-8. doi: 10.2106/JBJS.E.00320.*

The Authors thank the Reviewer for this comment, and are aware of these investigations, all of which reinforce the assertions made in the text (there is poor evidence for the necessity to reduce these fractures). All references noted above involve reduction, and randomization to different immobilization types or fixation types, except one which involves angulation (not displaced/translated injuries, our target injury). Addressing each: McLauchlan - randomized trial with no sample size calculations before study onset, no patient reported outcomes, and randomization between manipulation and long arm casting versus the same plus K-wire fixation. Colaris: similar to McLauchlan, all patients under age 16 were reduced and then randomized to K-wire fixation or continued long arm casting. No PROs. Seiler: all fractures reduced, all children under age 16, randomization to different immobilization types, no PROs. Adrian: angulated fractures, not under investigation herein, remodeling potential is fairly well established for angular deformity, also no PROs, only examiner ratings of outcome. Bohm: all fractures reduced, randomization to LAC vs SAC, all surrogate outcomes, no PROs. We have cited these studies in the Introduction and included the following: “Previous trials of distal radius fractures in children often involved surgical fixation, a focus on long arm vs. short arm casts, and none had patient reported outcomes.” (Page 5, Line 3)

4. *Although it is a pragmatic RCT, there is a need to describe the possible methods that will be used to carry out the two interventions evaluated. What are the possible types of plaster casts or orthoses that can be used? What will be the fracture reduction methods? Will rehabilitation be carried out?*

The pragmatic nature of the trial means that immobilization is not standardized, and treating physicians may immobilize in any way they wish (splint, cast of any type, any material, etc). Rehabilitation is not standardized nor under investigation. Fracture reduction includes closed reduction under the sedative protocols of the institution providing care. This pragmatic approach is a strength, since standardization of any of the above preclude its performance.

5. *Who will carry out the assessments and how will data be collected at 6 weeks and 3 months of follow-up (page 7, line 36)?*

Assessments of primary and almost all secondary outcomes are all patient reported outcomes (Table 1), administered directly to patients at the specified intervals via electronic surveys in automated fashion. These are also temporally unlinked from the follow-up visits with the treatment team. The secondary outcomes related to "revisions, reductions, complications" are all queried by research personnel in the medical record.

6. *What will be the criteria for fracture reducibility, loss of reduction and possible need for remanipulation or failure of reduction?*

The pragmatic nature of the trial involves complete freedom of the treating physician to accept fracture alignment as he/she sees fit. There are no criteria for reducibility, re-manipulation, and so on.
See response above under #4.

7. *How the secondary outcome Cosmesis will be measured (table 1; page 7, line 12)?*

   Cosmesis is measured by asking the parent to rate the appearance of the arm when completing the survey. They are given a sliding scale between 0 and 100 with 0 meaning "worst appearance possible" and 100 meaning "Normal – like an uninjured arm." We have added a brief description to the Outcomes section ("Cosmesis (assessed by parent rating of appearance)…" (Page 10, Line 24 – Page 11, Line 1)

8. *The radiographic outcome (page 11, line 41) is not included in Table 1. What will be the method and what radiographic parameters will be measured?*

   The Authors appreciate attention drawn to this point. Radiographic analysis has been added to the secondary outcomes portion of Table 2 (Page 12). Radiographs are only collected if part of standard of care, and no schedule is mandated. All radiographs will be collected as part of the study. Radiographic parameters will be reported by study personnel, including sagittal and coronal alignment by standard deformity analysis of the anatomic axis of the distal radius, as described by Paley et al. (Herzenberg, J. E., & Paley, D. (2005). *Principles of Deformity Correction* (1st ed.). Springer Berlin / Heidelberg. https://doi.org/10.1007/978-3-642-59373-4).

9. *The cost-effectiveness analysis method (page 13, line 3) needs to be clearly described, how and when it will be carried out?*

   The Authors appreciate attention brought to this point. No prospective cost data is being collected and this text has been removed from the manuscript text.

10. *What outcomes and how will they be measured in the analyzes carried out after one year of follow-up (page 13, line 3)?*

As follow-up beyond one year is dependent on securing additional funding. The protocol and corresponding SAP are limited to all analyses through one year. Additional SAPs will be developed to describe analytic plans for longer term follow-up. Briefly, we anticipate analyses will follow a similar modeling strategy using the mixed model framework. The Outcomes sections has been updated to reflect that exploratory outcomes beyond 1 year will be detailed elsewhere and are not part of this study design.

---

## VERSION 2 - REVIEW

---


| | |
|---|---|
| **Reviewer** | **3** |
| **Name** | **Launonen, Antti** |
| **Affiliation** | **Tampere University Hospital** |
| **Date** | **11-Dec-2024** |

## COI

Well improved manuscript. All the good for running the trial!

## VERSION 2 - AUTHOR RESPONSE

**Reviewer: 3**
*Dr. Antti Launonen, Tampere University Hospital*
*Comments to the Author:*
*Well improved manuscript. All the good for running the trial!*

We would like to express our gratitude for your helpful and supportive comments.

**Reviewer: 3**
*If you have selected 'Yes' above, please provide details of any competing interests.: Not applicable*

We have included details of any competing interests.
